# Examining bidirectional associations between cannabis use and internalizing symptoms among high-risk emerging adults: A prospective cohort study

**DOI:** 10.1017/S0033291725101700

**Published:** 2025-10-03

**Authors:** Jillian Halladay, Kyla Belisario, André McDonald, Samuel Acuff, Amanda Doggett, Molly Garber, Andrea Maxwell, James Murphy, James MacKillop

**Affiliations:** 1 School of Nursing, McMaster University, Hamilton, ON, Canada; 2 Peter Boris Centre for Addictions Research, McMaster University/St. Joseph’s Healthcare, Hamilton, ON, Canada; 3 Department of Psychiatry and Behavioural Neurosciences, McMaster University, Hamilton, ON, Canada; 4 Michael G. DeGroote Centre for Medicinal Cannabis Research, McMaster University, Hamilton, ON, Canada; 5 Recovery Research Institute, Department of Psychiatry, Massachusetts General Hospital and Harvard Medical School, Boston, MA, USA; 6 Department of Psychology, Neuroscience, and Behaviour, McMaster University, Hamilton, ON, Canada; 7 Department of Psychology, University of Memphis, Memphis, TN, USA

**Keywords:** anxiety, bidirectional relationships, cannabis, comorbidity, cross-lagged, depression, longitudinal, prospective

## Abstract

**Background:**

The co-occurrence of cannabis use and internalizing symptoms, such as depression and anxiety, during emerging adulthood (18–25 years) is well documented. However, while bidirectional relationships are often assumed, empirical evidence is mixed. This study investigates bidirectional longitudinal relationships between cannabis frequency and consequences and internalizing symptoms (depressive and anxiety) among high-risk emerging adults.

**Methods:**

Data came from seven assessments collected over a 2-year period among 961 (54% female) high-risk emerging adults participating in two longitudinal cohorts (Ontario, Canada; Tennessee, USA). Assessments were at 4-month intervals spanning 2018–2020. Latent curve models with structured residuals were used to explore bidirectional between- and within-person relationships between cannabis-related variables and internalizing symptoms.

**Results:**

At baseline, higher levels of cannabis frequency and consequences were associated with higher internalizing symptoms. In between-person model components, cannabis-related and internalizing variables decreased across emerging adulthood. Significant within-person bidirectional relationships were observed, partially supporting both symptom-driven and substance-induced pathways, but the findings were specific to negative cannabis consequences, not frequency, and for depressive symptoms, not anxiety symptoms, for symptom-driven pathways. These bidirectional relationships were more pronounced among females and those surpassing clinical thresholds for internalizing symptoms at baseline.

**Conclusions:**

This study found evidence of bidirectional relationships between cannabis consequences and internalizing symptoms across emerging adulthood, with the prevailing direction from cannabis-related negative consequences to increases in internalizing symptoms. These findings highlight the importance of cannabis intervention in emerging adults, both to reduce consequences and to prevent internalizing disorders, especially targeting females and those with clinically elevated internalizing symptoms.

## Introduction

The co-occurrence of cannabis use and internalizing problems (here defined as depression and anxiety) among adults has been increasing over the past two decades (Gorfinkel, Stohl, & Hasin, 2020; Halladay, Munn, Boyle, Jack, & Georgiades, 2019b; Han, Compton, Einstein, & Volkow, 2021; Pacek, Weinberger, Zhu, & Goodwin, 2020). Cannabis is one of the most commonly used substances, and internalizing problems are the most common type of mental health concern, both peaking during emerging adulthood (ages 18–25; Health Canada, 2023; Solmi et al., 2022). Cannabis use tends to decrease during emerging adulthood (Cristiano & Sharif-Razi, 2019; Lee & Sher, 2018). However, recent trends show increasing rates of cannabis use that are particularly pronounced among emerging adults (Caulkins, 2024; Hall, Stjepanović, Dawson, & Leung, 2023). While some research suggests that internalizing problems also decrease across emerging adulthood and into adulthood (Blanchflower, 2021), other studies have found that, after accounting for birth cohort, symptoms remain elevated and continue increasing with age (Halladay et al., 2023a). Moreover, internalizing problems have spiked among young people from recent birth cohorts (born 1980 onward; Halladay, Slade, et al., 2023a). Given these trends, it is important to note that cannabis and internalizing problems commonly co-occur, and their co-occurrence has been linked to more severe and longer-term problems (Gobbi et al., 2019; Lev-Ran et al., 2014; Moitra, Christopher, Anderson, & Stein, 2015). While bidirectional relationships are often assumed, empirical evidence related to the specific reasons and pathways to their co-occurrence remain unclear (Garey et al., 2020; Kuhns, Kroon, Colyer-Patel, & Cousijn, 2022). As cannabis use and internalizing problems increase at the population level, understanding their co-development at the person-level is crucial for informing public health messaging, prevention strategies, and effective early interventions.

There are three common hypotheses discussed in the context of co-occurring substance use and internalizing problems. First, the *symptom-driven hypothesis* posits that internalizing problems lead to the onset or escalation of substance use, often attributed to using substances for coping purposes (Moitra et al., 2015). Second, the *substance-induced hypothesis* indicates that substance use leads to the onset or worsening of internalizing problems due to neurobiological or psychosocial mechanisms (Gobbi et al., 2019; Lev-Ran et al., 2014). Third, the *shared vulnerability hypothesis* suggests substance use and internalizing problems co-occur because of shared risk and protective factors (Vanyukov & Ridenour, 2012). These hypotheses are not meant to be mutually exclusive. Some evidence shows bidirectional relationships between cannabis use and internalizing problems where both symptom-driven and substance-induced pathways occur simultaneously (Lydiard et al., 2023). The nature and strength of these relationships has also been found to vary depending on what factors are controlled for in the analysis due to the influence of shared vulnerability (Garey et al., 2020; Halladay, 2024). Identifying directional pathways between cannabis and internalizing problems can help inform the timing and targets for policy, prevention, and intervention initiatives.

Evidence regarding the strength and direction of relationships between cannabis use and internalizing problems remains inconsistent (Garey et al., 2020; Kuhns et al., 2022). Mixed results may, in part, be due to study differences in the types of internalizing symptoms, operationalization of cannabis use, developmental age periods, and/or analytical approaches explored. First, associations between internalizing symptoms and cannabis are generally stronger and more consistently positive for depression than anxiety (Amendola, Hengartner, Ajdacic-Gross, Angst, & Rössler, 2022; Colder, Lee, Frndak, Read, & Wieczorek, 2019; Duperrouzel et al., 2018; Gobbi et al., 2019; London-Nadeau et al., 2021). This suggest that while various internalizing disorders such as depression and anxiety commonly co-occur, they are distinct constructs and relations between cannabis and specific internalizing problems may be distinct. Second, directional relationships may differ depending on whether cannabis use or cannabis problems/consequences are examined. Cannabis problems/consequences appear more robustly associated with internalizing symptoms than frequency of use (Kuhns et al., 2022). This is not surprising given that some consequence items refer to experiences that are likely influenced both by cannabis use and more general affective states (e.g., I have been unhappy because of my cannabis use). Third, the specific pathways to co-occurrence may be developmentally driven, with directional pathways changing across adolescent and emerging adult development. Prior research shows more pronounced symptom-driven pathways among adolescents (Bolanis et al., 2020; Colder et al., 2019; London-Nadeau et al., 2021; Rhew et al., 2017), while substance-driven pathways are more pronounced among emerging adults (Davis et al., 2022, 2023a, 2023b; Wang et al., 2022; Womack, Shaw, Weaver, & Forbes, 2016). Fourth, historical analytical models for exploring bidirectional relationships (e.g., cross-lagged panel models) have been found to have methodological limitations, though there are new contemporary methods that better disentangle bidirectional differences (Littlefield et al., 2022). Fifth, time-lags between assessments may impact results, with most prior work exploring 1-year lags between assessments which may be masking directional relationships on a shorter time scale.

Despite the higher prevalence of substance use disorders among males, females experience more internalizing problems (McGrath et al., 2023) and are more likely to transition from substance use to substance use problems (Bassir Nia, Mann, Kaur, & Ranganathan, 2018) than males. Females also tend to experience a higher prevalence of co-occurring problems than males (Halladay, Boyle, Munn, Jack, & Georgiades, 2019a; Kuhns et al., 2022). Some prior work has explored sex differences in developmental relationships between cannabis use and internalizing symptoms. In a community sample of US emerging adults, stronger bidirectional relationships between cannabis frequency and anxiety symptoms were found among females compared to males (Davis et al., 2022). However, in the same sample, bidirectional effects were more pronounced among males when exploring pathways between cannabis frequency and post-traumatic stress disorder (PTSD) symptoms (Davis et al., 2023b). In a different sample of US emerging adults, sex did not moderate developmental pathways between cannabis frequency and anxiety or depressive symptoms (Colder et al., 2019). As such, sex-specific directional pathways between cannabis and specific internalizing disorders remain unclear and understudied.

Most who use cannabis will not experience significant problems, though individuals who also experience symptoms of depression or anxiety are at heightened risk of negative consequences from use (Fischer et al., 2017). Notably, young people accessing treatment for cannabis use problems often report high levels of internalizing symptoms, with many having previously accessed mental health services for problems other than substance use (Halladay et al., 2023b; Hawkins, 2009; Turner, Muck, Muck, Stephens, & Sukumar, 2004). A previous cross-lagged panel analysis exploring connections between cannabis use and depressive symptoms among a sample of young men found that cannabis at age 17 predicted higher depressive symptoms at age 20, but only among those who had clinically elevated depressive symptoms during the period of observation (Womack et al., 2016). As such, clinically elevated internalizing symptoms may contribute to differential patterns (magnitude and/or direction) of relationships between cannabis use/consequences and internalizing problems across development.

This study seeks to comprehensively explore the between- and within-person longitudinal bidirectional relationships across emerging adulthood. Data come from two longitudinal cohorts of high-risk emerging adults in Hamilton, Ontario (Canada) and Memphis, Tennessee (USA). By applying contemporary analytical approaches, this study explores whether cannabis use consequences and/or frequency are associated with depression and/or anxiety symptoms over time. Secondly, this study explores whether these relationships are moderated by sex assigned at birth or being above clinical thresholds for internalizing measures at baseline. At the between-person level, we hypothesized that higher cannabis use and related consequences at baseline would be associated with higher internalizing symptoms at baseline (correlated intercepts) and that change over time (slopes) between both domains would be correlated. At the within-person level, we hypothesized that there would be significant bidirectional relationships over time noted by positive cross-lagged effects in both directions. We additionally hypothesized that there would be stronger relationships for females (Halladay, Boyle, et al., 2019a) and those with a clinically meaningful internalizing symptom score at baseline (Womack et al., 2016).

## Methods

### Data

This is a secondary analysis of data from two independent longitudinal cohorts of emerging adults who were recruited from the community in Memphis, Tennessee (Baseline *N* = 601) and Hamilton, Ontario (Baseline *N* = 730), combined into a single sample. The parent studies are called the Behavioral Economic Trajectories of Alcohol Misuse Hamilton (BETA-H) and Memphis (BETA-M) studies. Prior publications using this data, alongside our unique hypotheses and analysis plans, were pre-registered: https://doi.org/10.17605/OSF.IO/SDM4K.

In both samples, waves of data collection began on a rolling basis in 2017 with in-person assessments, and follow-up assessments occurred every 4 months (in-person or online assessments). Assessments were re-organized using 4-month intervals, with a longer time span for the first time point due to the rolling nature of enrollment into both studies (up until March 10th, 2018). This study used 7 time points spanning 2018–2020; no assessments were included after the declaration of COVID-19 as a global pandemic by the WHO (March 11th, 2020).

Participants were recruited through ads in newspapers, online, email listservs, universities, and at bus stops. Eligibility for both cohorts included (1) regular heavy episodic drinking, defined as more than four standard drinks (males) or three standard drinks (females) in a single drinking occasion on 3 or more days in the past month, (2) fluency in English, (3) no current or past psychotic disorders, and (4) within the target age range (i.e., 21.5–24.9, Memphis sample; 19.5–<23, Hamilton sample). Eligibility for the Canadian sample also permitted two instances of heavy episodic drinking in the past month in conjunction with a participant also reporting using cannabis at least once per month. The difference in minimum age reflected differences in the legal drinking age between Ontario, Canada (19 years), and the United States (21 years). Further, for the main analysis, only participants who endorsed past-month cannabis use for at least one wave (72.2% of total sample) were included given our interest in relationships between variability in cannabis frequency/problems with internalizing symptoms. Including the full sample would introduce a large subgroup (~33%) with no variability in cannabis use, potentially attenuating associations and biasing estimates downward. Participants were also required to demonstrate adequate attention/effort based on quality control measures, which was assessed throughout by administering several non-ambiguous questions with one correct response (e.g., ‘For this item, please choose “Never”’). The final combined sample size was *N* = 961 (See Supplementary Materials [SM] for details). Of these participants, 93.7% had more than two waves of data (Mean ± SD = 5.58 ± 1.51; Median = 6). To facilitate comparisons with other analyses that do not separate out those using cannabis from those who do not, we conducted additional analyses using the total sample (99.6% of total sample; n = 1,331).

### Measures

#### Cannabis variables

Cannabis use frequency was assessed using the Alcohol, Smoking, and Substance Involvement Screening Test measure (WHO ASSIST Working Group, 2002). Participants were asked, ‘*What was your typical cannabis use during the last month?’* with response options: none (0), monthly (1), weekly (2), daily (3), or multiple times daily (4). Note that participants were asked to select ‘monthly’ if they had only used once. Cannabis frequency was treated ordinally.

Cannabis-related consequences were measured using the Brief Marijuana Consequences Questions (Simons, Dvorak, Merrill, & Read, 2012). This is a 21-item measure with questions answered with ‘Yes’ or ‘No’ regarding social or interpersonal, academic or occupational, self-perception, risky behaviors, self-care, blackout, impaired control, and physiological dependence related consequences. Items are preceded by the statement, ‘The following is a list of things that sometimes happen to people either during, or after they have been using marijuana’. Items were summed from 0–21, where higher scores reflect more consequences. Cannabis consequences was treated continuously (Cronbach’s alpha mean [min–max] across waves = 0.89 [0.87–0.91]).

#### Internalizing variables

Symptoms of depression were measured with the 9-item Patient Health Questionnaire (PHQ-9) that asks about the frequency of depressive symptoms over the past month (Kroenke, Spitzer, & Williams, 2001). Items relate to difficulties with mood, anhedonia, sleep, energy, appetite, concentration, movements, and suicidality (a = 0.90 [0.89–0.91]). Items were summed from 0–27, where higher scores reflect worse symptoms; scores > = 10 are indicative of moderate to severe depressive symptoms (Kroenke et al., 2001).

Symptoms of anxiety were measured with the 7-item Generalized Anxiety Disorder measure (GAD-7) which asks about the frequency of anxiety symptoms over the past month (Spitzer, Kroenke, Williams, & Löwe, 2006). Items ask about feeling nervous, unable to control worry, worrying about many different things, trouble relaxing, restlessness, irritability, and fear (a = 0.93 [0.92–0.94]). Items were summed from 0 to 21, where higher scores reflect worse symptoms, with scores > = 10 reflecting moderate to severe anxiety symptoms (Spitzer et al., 2006).

#### Confounders

Between-person time-invariant confounders included: age, sex assigned at birth (reference male), race (Asian, Black, Caucasian, Other; reference Caucasian), sexual orientation (reference heterosexual), educational status (enrollment in or completion of 4-year degree; reference less than 4 year degree), ACEs (Alhowaymel, Kalmakis, Chiodo, Kent, & Almuneef, 2023; Felitti et al., 1998; reference score < 4), and family history of substance use problems (CAST score; Hodgins, Maticka-Tyndale, El-Guebaly, & West, 1993; reference those without reported parental alcohol or drug problems). Within-person time-varying confounders included total drinks per week (measured by the Daily Drinking Questionnaire; Collins, Parks, & Marlatt, 1985), nicotine use (determined by the maximum frequency of cigarette or e-cigarette use endorsed; WHO ASSIST Working Group, 2002), and subjective household financial situation (Najdzionek, McIntyre-Wood, Amlung, & MacKillop, 2023).

#### Moderators

Moderators of primary interest to the current analysis include sex assigned at birth (56% Female, 44% Male) and clinical cut-off scores of 10+ on either PHQ-9 (34%) or GAD-7 (25%) at baseline. We also explored moderation related to site location (60% Hamilton, 40% Memphis) as a sensitivity analysis.

Of note, literature suggests other potential nuanced subgroup differences in bidirectional relationships between cannabis and internalizing symptoms based on: sexual orientation (Felner et al., 2020; London-Nadeau et al., 2021; McCabe et al., 2020), adverse traumatic childhood experiences (Davis et al., 2023a; Grummitt, Baldwin, Lafoa’i, Keyes, & Barrett, 2024; Pagano et al., 2007), and family history (Trucco, 2020). As such, these moderators were of secondary interest with details and results reported in SM following pre-registered analyses.

### Statistical analysis

The modelling technique used was latent curve models with structured residuals (LCM-SR; Curran, Howard, Bainter, Lane, & McGinley, 2014). This is an extension of the latent growth curve model, which includes lagged effects of the structured residuals, allowing for simultaneous consideration of both person-specific and time-specific influences. In these models, the latent factors capture interindividual differences in intraindividual change over time (Curran et al., 2014), allowing for ease of interpretation of the mean structure since the between-person and within-person aspects are separated in the model (Andersen, 2022).

We ran a series of models (with increasing covariate adjustments) for four combinations of cannabis variables (frequency and consequences) and internalizing variables (depressive and anxiety symptoms). For each combination, the following main models were run: (1) unadjusted main effects model; (2) sociodemographic between-person confounders added to Model 1; and (3) other between-person confounders added to Model 2 (main model); and (4) time-varying confounders added to Model 3^1^. Model 3 was repeated with each stratification variable as an effect modifier^2^. Model 3 main effect results are presented in the main manuscript. We also conducted two post-hoc sensitivity analyses whereby the main models (Model 3) were re-estimated using: (a) the full sample without any cannabis use exclusions, and (b) a reduced cannabis consequences measures dropping items attributing cannabis use to mental health. Additional results are available in SM. The PHQ-9 and GAD-7 were pro-rated (i.e., within-person mean imputation) for one missing item. Any further missingness in cannabis or internalizing outcomes were handled with Full Information Maximum Likelihood, a method for handling missingness in structural equation modelling frameworks that produces unbiased estimates when data are missing at random (Enders & Bandalos, 2001).

As cannabis frequency was modelled ordinally, these models were fit using the weighted least square mean and variance adjusted estimator. Cannabis consequences were modelled continuously, and thus these models used the robust maximum likelihood estimator. Several model fit indices were examined to determine the best-fitting model components (function of time and within-person effects) before combining as one LCM-SR model (Curran et al., 2014). To determine the function of time, non-growth, linear growth, and quadratic growth model fit statistics (AIC, SA-BIC, CFI, TLI, RMSEA) were compared, and the best-fitting function of time with statistically significant growth factors was retained. To determine the best modelling of the within-person effects (the cross-lagged panel model portion of the model), model selection was based on χ2 difference testing (or Satorra–Bentler scaled χ2 difference) since the models are inherently nested (Satorra & Bentler, 2001). In final models, for the within-person effects, the standardized effects can be interpreted as the following effect sizes: 0.03 (small); 0.07 (medium); 0.12 (large) (Orth et al., 2024). We inferred statistical significance where p-values were < 0.05. Notably, statistical power was tested using a Monte Carlo simulation in Mplus (Version 8.10) with an expected power of 0.943 for cross-lagged effects when estimating a small effect of 0.03 (See pre-registration for details).

The presence of significant modifiers was tested by fitting two models; one which constrained the within- and between-components equal across the stratifying variable, and one which allowed these components to vary, with the presence of modifiers determined by χ2 difference testing (or Satorra–Bentler scaled χ2 difference). Notably, moderation effects were assessed on: (1) whether the within- and between-components could be constrained equal across groups, and if not, (2) whether there were meaningful patterns of results in terms of both significance and magnitude of effects which distinguish one group from the other.

## Results

### Overall trends

**Figure 1.**
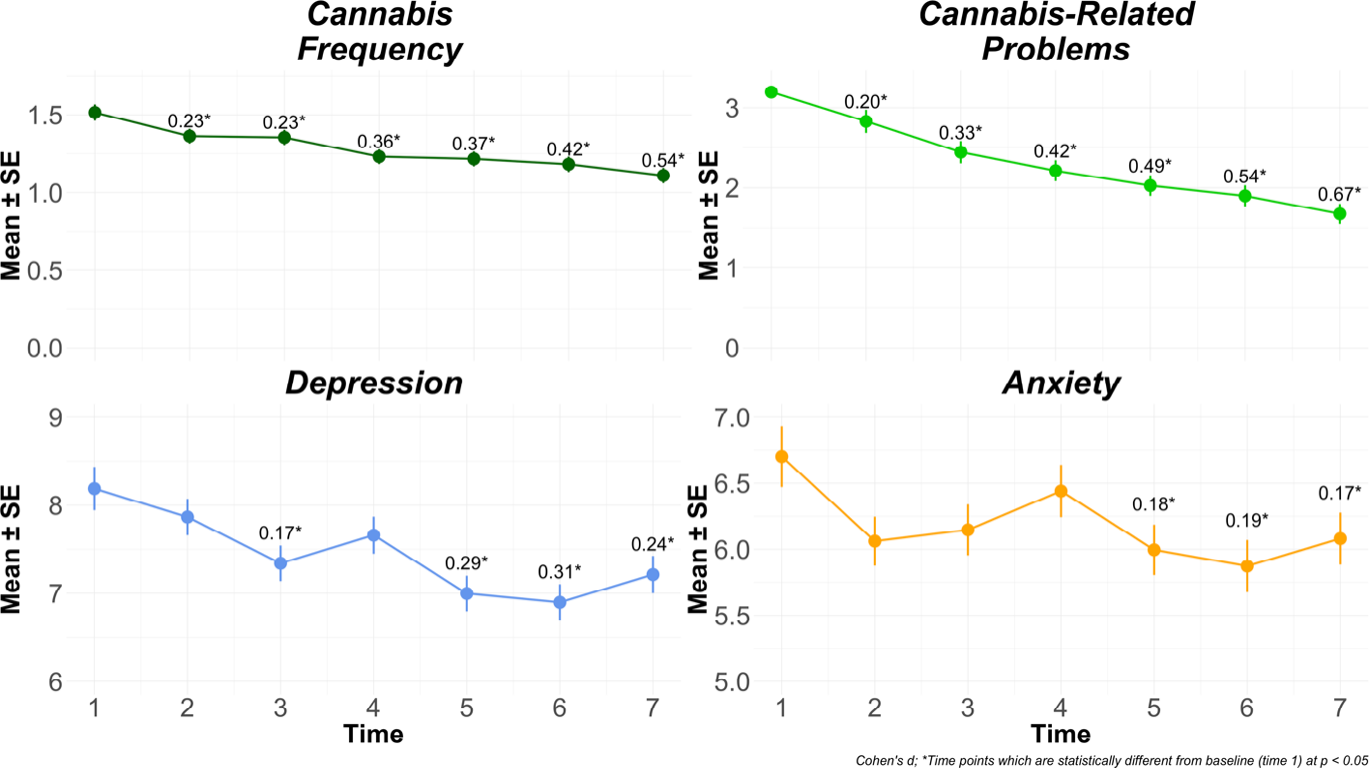
Overall trends (panel). *Caption:* These figures show the average cannabis or internalizing symptom scores across time points. The values above each average indicate the Cohen’s d effect size between the specified time point and baseline.

**Figure 2.**
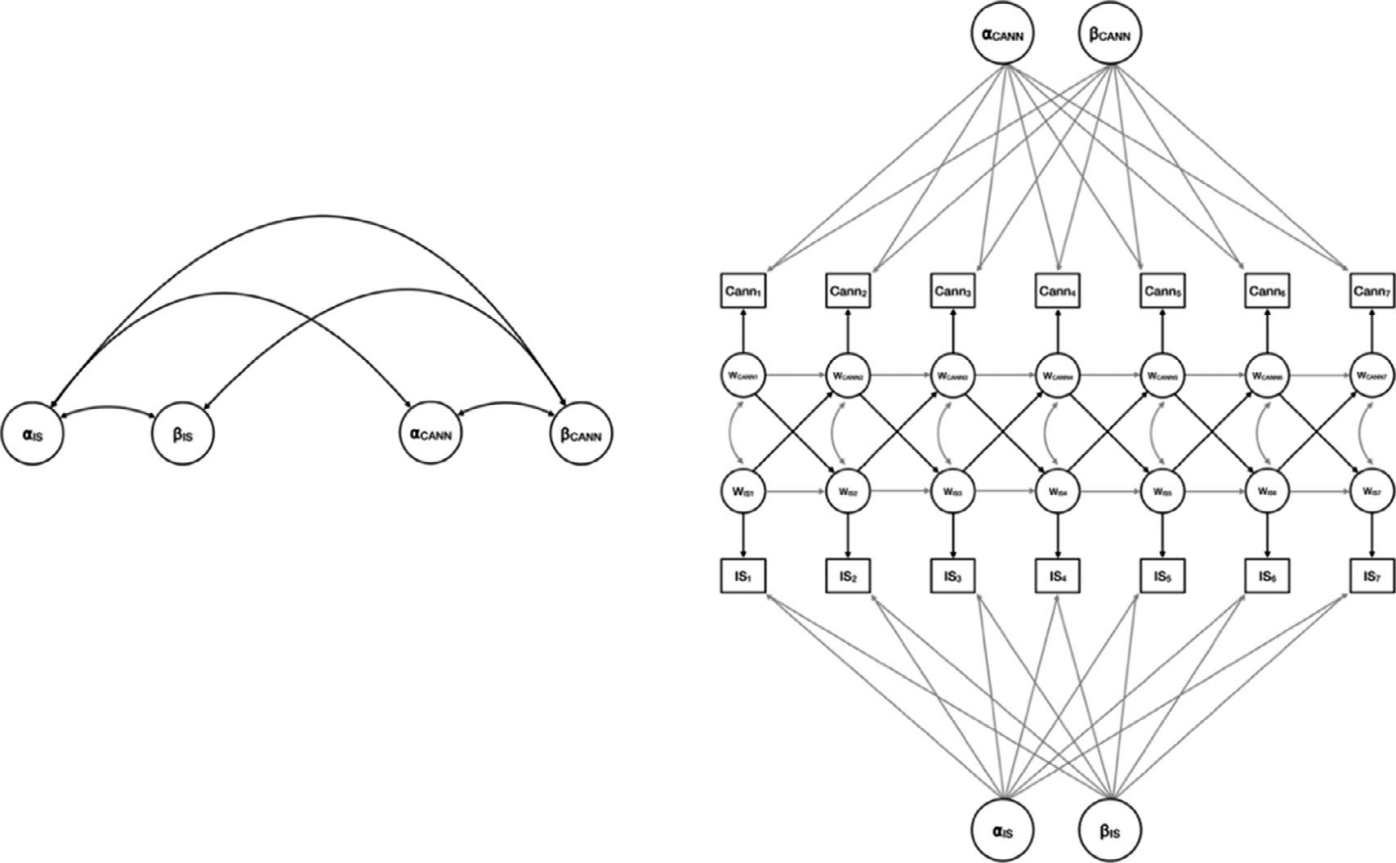
Final modeling structure. *Caption*: Final models were linear, with fully constrained within-person effects. The left side of the figure depicts the between-person model. The right side of the figure depicts the within-person model. Here, *a*, ‘intercepts’; *β*, ‘slopes’; w, ‘wave’; IS, ‘internalizing symptoms’; CANN, ‘cannabis variables’.

See Table 1 for descriptive characteristics of the sample. Overall descriptive trends for cannabis frequency, cannabis consequences, depressive symptoms, and anxiety symptoms demonstrated decreases across emerging adulthood (See Figure 1). The best-fitting models were those with a linear function of time and fully constrained within-person effects (See SM and Figure 2). Given that within-person effects are constrained across waves, average effects across waves from Model 3 are presented in the text (See Table 2 for specific effects). The unadjusted, sociodemographic-adjusted, and other time-invariant and varying confounder-adjusted models yielded similar results (See SM).

**Table 1.** Descriptive characteristics overall and by sex assigned at birth

Characteristic	Overall Sample*N* = 961	Female*N* = 517	Male*N* = 444	p-value
Sample				0.3
*Hamilton*	574 (59.73%)	301 (58.22%)	273 (61.49%)	
*Memphis*	387 (40.27%)	216 (41.78%)	171 (38.51%)	
Age	21.89 +/− 1.25	21.86 +/− 1.24	21.91 +/− 1.26	0.5
Race				<0.001
*Black*	180 (18.73%)	121 (23.40%)	59 (13.29%)	
*Asian*	110 (11.45%)	57 (11.03%)	53 (11.94%)	
*Other*	99 (10.30%)	56 (10.83%)	43 (9.68%)	
*White*	572 (59.52%)	283 (54.74%)	289 (65.09%)	
Sexual Orientation				<0.001
*Heterosexual*	686 (71.38%)	330 (63.83%)	356 (80.18%)	
*2SLGBTQIA+*	275 (28.62%)	187 (36.17%)	88 (19.82%)	
Subjective Income				<0.001
*Not Enough*	49 (5.10%)	40 (7.74%)	9 (2.03%)	
*Cut Back*	269 (27.99%)	155 (29.98%)	114 (25.68%)	
*No Extras*	301 (31.32%)	158 (30.56%)	143 (32.21%)	
*Enough for Extras*	342 (35.59%)	164 (31.72%)	178 (40.09%)	
4-Year Educational Outcome				0.5
*Less than a Bachelors*	127 (13.22%)	70 (13.54%)	57 (12.84%)	
*Bachelors or Higher*	834 (86.78%)	447 (86.46%)	387 (87.16%)	
Cannabis ASSIST (Frequency)^a^				<0.001
*None*	195 (20.31%)	131 (25.39%)	64 (14.41%)	
*Monthly*	353 (36.77%)	197 (38.18%)	156 (35.14%)	
*Week*	184 (19.17%)	81 (15.70%)	103 (23.20%)	
*Daily*	103 (10.73%)	44 (8.53%)	59 (13.29%)	
*Daily+*	125 (13.02%)	63 (12.21%)	62 (13.96%)	
MACQ-Brief^a^ Score	3.33 +/− 4.19	2.89 +/− 3.95	3.84 +/− 4.40	<0.001
CUDIT Score^a^	7.03 +/− 6.74	6.23 +/− 6.67	7.97 +/− 6.71	<0.001
PHQ–9 Score^a^	7.98 +/− 5.81	8.96 +/− 6.04	6.84 +/− 5.32	<0.001
GAD–7 Score^a^	6.49 +/− 5.42	7.53 +/− 5.77	5.29 +/− 4.71	<0.001

a Missingness: Cannabis ASSIST missing n = 1; MACQ-Brief missing n = 4; CUDIT missing n = 2; PHQ-9 missing n = 3; GAD-7 missing n = 4.

**Table 2.** Main effects models (Model 3; n = 961)^a^

	Depressive Symptoms		Anxiety Symptoms	
	Frequency	Consequences	Frequency	Consequences
Within-Person: Cannabis to Internalizing				
T1➔ T2	0.003 (0.04) p > 0.9	**0.093 (0.04) p = 0.016**	0.056 (0.04) p = 0.160	**0.077 (0.04) p = 0.043**
T2 ➔ T3	0.003 (0.04) p > 0.9	**0.086 (0.04) p = 0.018**	0.050 (0.04) p = 0.159	**0.066 (0.03) p = 0.043**
T3 ➔ T4	0.003 (0.03) p > 0.9	**0.074 (0.03) p = 0.017**	0.046 (0.03) p = 0.157	**0.061 (0.03) p = 0.038**
T4 ➔ T5	0.003 (0.04) p > 0.9	**0.082 (0.04) p = 0.020**	0.052 (0.04) p = 0.162	**0.069 (0.03) p = 0.040**
T5 ➔ T6	0.003 (0.04) p > 0.9	**0.079 (0.03) p = 0.018**	0.050 (0.04) p = 0.162	**0.060 (0.03) p = 0.038**
T6 ➔ T7	0.003 (0.04) p > 0.9	**0.086 (0.04) p = 0.023**	0.055 (0.04) p = 0.160	**0.068 (0.03) p = 0.044**
Within-Person: Internalizing to Cannabis				
T1 ➔ T2	0.001 (0.03) p > 0.9	**0.053 (0.03) p = 0.040**	0.02 (0.03) p = 0.549	0.029 (0.02) p = 0.226
T2 ➔ T3	0.001 (0.03) p > 0.9	**0.055 (0.03) p = 0.042**	0.02 (0.03) p = 0.549	0.030 (0.03) p = 0.234
T3 ➔ T4	0.001 (0.04) p > 0.9	**0.055 (0.03) p = 0.042**	0.02 (0.04) p = 0.550	0.033 (0.03) p = 0.232
T4 ➔ T5	0.001 (0.04) p > 0.9	**0.066 (0.03) p = 0.041**	0.03 (0.04) p = 0.550	0.036 (0.03) p = 0.230
T5 ➔ T6	0.001 (0.04) p > 0.9	**0.057 (0.03) p = 0.033**	0.02 (0.04) p = 0.549	0.031 (0.03) p = 0.225
T6 ➔ T7	0.001 (0.03) p > 0.9	**0.058 (0.03) p = 0.045**	0.02 (0.04) p = 0.549	0.035 (0.03) p = 0.240
Within-Person: Cannabis to Cannabis				
T1➔ T2	**0.165 (0.04); p < 0.001**	**0.242 (0.07); p < 0.001**	**0.169 (0.04); p < 0.001**	**0.247 (0.07); p < 0.001**
T2 ➔ T3	**0.167 (0.04); p < 0.001**	**0.235 (0.07); p < 0.001**	**0.172 (0.04); p < 0.001**	**0.241 (0.07); p < 0.001**
T3 ➔ T4	**0.167 (0.04); p < 0.001**	**0.224 (0.06); p < 0.001**	**0.172 (0.04); p < 0.001**	**0.229 (0.07); p < 0.001**
T4 ➔ T5	**0.167 (0.04); p < 0.001**	**0.245 (0.07); p < 0.001**	**0.172 (0.04); p < 0.001**	**0.251 (0.07); p < 0.001**
T5 ➔ T6	**0.167 (0.04); p < 0.001**	**0.213 (0.06); p < 0.001**	**0.172 (0.04); p < 0.001**	**0.218 (0.06); p < 0.001**
T6 ➔ T7	**0.167 (0.04); p < 0.001**	**0.239 (0.07); p < 0.001**	**0.172 (0.04); p < 0.001**	**0.245 (0.07); p < 0.001**
Within-Person: Internalizing to Internalizing				
T1➔ T2	**0.082 (0.02); p < 0.001**	0.038 (0.04); p = 0.360	**0.200 (0.03); p < 0.001**	**0.136 (0.04); p < 0.001**
T2 ➔ T3	**0.085 (0.02); p < 0.001**	0.037 (0.04); p = 0.375	**0.181 (0.02); p < 0.001**	**0.128 (0.04); p < 0.001**
T3 ➔ T4	**0.075 (0.02); p < 0.001**	0.034 (0.04); p = 0.365	**0.193 (0.03); p < 0.001**	**0.134 (0.04); p < 0.001**
T4 ➔ T5	**0.099 (0.03); p < 0.001**	0.041 (0.05); p = 0.369	**0.232 (0.03); p < 0.001**	**0.155 (0.04); p < 0.001**
T5 ➔ T6	**0.087 (0.03); p < 0.001**	0.039 (0.04); p = 0.360	**0.202 (0.03); p < 0.001**	**0.129 (0.03); p < 0.001**
T6 ➔ T7	**0.091 (0.027); p = 0.001**	0.039 (0.04); p = 0.374	**0.228 (0.04); p = 0.000**	**0.150 (0.04); p < 0.001**
Between-Person: Cannabis Intercept with				
Internalizing Intercept	**0.242 (0.06) p < 0.001**	**0.276 (0.05) p < 0.001**	**0.203 (0.06) p = 0.001**	**0.234 (0.06) p < 0.001**
Cannabis Slope	**−0.451 (0.06) p < 0.001**	**−0.638 (0.09) p < 0.001**	**−0.449 (0.06) p < 0.001**	**−0.637 (0.09) p < 0.001**
Internalizing Slope	−0.134 (0.10) p = 0.174	−0.055 (0.09) p = 0.541	−0.038 (0.12) p = 0.740	0.065 (0.11) p = 0.559
Between-Person: Cannabis Slope with				
Internalizing Intercept	−0.131 (0.08) p = 0.102	−0.078 (0.11) p = 0.478	−0.115 (0.08) p = 0.149	−0.073 (0.12) p = 0.552
Internalizing Slope	0.217 (0.15) p = 0.144	0.081 (0.20) p = 0.688	0.098 (0.17) p = 0.561	−0.036 (0.25) p = 0.887
Between-Person: Internalizing Intercept with				
Internalizing Slope	**−0.336 (0.07) p < 0.001**	**−0.385 (0.07) p < 0.001**	**−0.296 (0.09) p = 0.001**	**−0.328 (0.10) p = 0.001**

a Adjusted for between-person effects of age, sex, race, sexual orientation, degree, trauma, and family history.

### Cannabis and depressive symptoms

Between-person effects indicated that those who began the study with higher levels of cannabis frequency or consequences also started with higher depressive symptoms (β_STD_ = 0.24 and β_STD_ = 0.28, respectively). Those with higher initial cannabis frequency or consequences experienced larger declines in these measures (β_STD_ = −0.45 and β_STD_ = −0.64). Similarly, those with higher initial depressive symptoms experienced larger declines in depressive symptoms over time (β_STD_ = −0.34 and β_STD_ = −0.38). However, *changes* in cannabis frequency or consequences and depressive symptoms (slopes) were not significantly correlated between persons.

Within-person effects showed significant autocorrelation for cannabis frequency and consequences and for depressive symptoms in the cannabis frequency models (i.e., cannabis at time *T* predicted cannabis at time *T* + 1). There were no significant within-person cross-lagged effects found in either direction for cannabis frequency and depressive symptoms. There were significant cross-lagged effects for cannabis consequences predicting subsequent depressive symptoms (e.g., averaged time-lags β_STD_ = 0.083, medium-large effect) and for depression predicting subsequent cannabis consequences (e.g., averaged β_STD_ = 0.06, small-medium effect). Similar significant effects and effects sizes were found in the full sample, regardless of cannabis use (See SM; Cannabis Consequences to Depression: β_STD_ = 0.06–0.08; p-values = 0.016–0.023; Depression to Cannabis Consequences: β_STD_ = 0.04–0.06; p-values = 0.0313–0.040). The overall pattern of results remained consistent when adjusting for time-varying covariates. Similar direction and significance of effects remained when adjusted for time-varying smoking or income (See SM). However, while the pattern was similar, the significance of effects differed when accounting for time-varying alcohol use. Specifically, significant bidirectional effects emerged between cannabis frequency and depressive symptoms (Cannabis Frequency to Depression: β_STD_ = 0.16–0.19, p-values <0.001; Depression to Cannabis Frequency β_STD_ = 0.13–0.15, p-values <0.001), while relationships between depressive symptoms and cannabis consequences became unidirectional (Cannabis Consequences to Depression: β_STD_ = 0.07–0.09, p-values = 0.023–0.032; Depression to Cannabis Consequences: β_STD_ = 0.05–0.06, p-values = 0.045–0.058). Further, when removing consequences adjacent to internalizing symptoms (e.g., I have been unhappy because of my marijuana use), significant cross-lags where depression predicted subsequent cannabis consequences remained (β_STD_ = 0.06–0.07; p-values = 0.026–0.036) while the reverse cross-lags became non-significant (β_STD_ = 0.05–0.07; p-values = 0.071–0.083).

### Cannabis and anxiety symptoms

Between-person effects indicated that those who began the study with higher levels of cannabis frequency and consequences also had higher anxiety symptoms at baseline (β_STD_ = 0.20 and β_STD_ = 0.23, respectively). Those who started the study with higher cannabis frequency or consequences experienced larger declines in these measures over time (β_STD_ = −0.45 and β_STD_ = −0.64). Similarly, those with higher initial anxiety symptoms experienced larger declines in symptoms over time (β_STD_ = −0.30 and β_STD_ = −0.33). However, *changes* in cannabis frequency or consequences and anxiety symptoms (slopes) were not significantly correlated between persons.

Within-person effects show significant autocorrelation for cannabis frequency, cannabis consequences, and anxiety symptoms. There were no significant cross-lagged effects found for cannabis frequency and anxiety symptoms in either direction, but significant cross-lagged effects were found for cannabis consequences predicting subsequent anxiety symptoms (e.g., averaged β_STD_ = 0.068, medium effect), though not for anxiety symptoms predicting subsequent cannabis consequences. Similar significant within-person cross-lagged effects were found in the full sample (Cannabis Consequences to Anxiety: β_STD_ = 0.06–0.07; p-values = 0.035–0.041) and when using the cannabis consequences measure with internalizing-adjacent items removed (Cannabis Consequences to Anxiety: β_STD_ = 0.06; p-values = 0.043–0.049). Patterns of results were also similar when adjusting for time-varying covariates, though cross-lags became non-significant when adjusting for time-varying alcohol (Cannabis Consequences to Anxiety: β_STD_ = 0.06–0.07; p-values = 0.055–0.62) but became bidirectionally significant between cannabis consequences and anxiety symptoms when adjusting for time-varying smoking or income (See SM).

### Subgroup differences

For cannabis frequency, models were invariant based on sex-assigned at birth and across internalizing clinical thresholds. For cannabis consequences, sex-assigned at birth and internalizing clinical cut-offs yielded significant subgroup differences. First, cannabis consequences were more strongly related to internalizing symptoms for females compared to males (See Table 3). Among females, there were significant bidirectional cross-lagged effects for both depressive and anxiety symptoms, while males had no notable cross-lagged effects. Second, cannabis consequences were more strongly related to depressive symptoms among those *above* clinical cut-offs (See Table 4). Specifically, those above depression clinical cut-offs at baseline had medium–large bidirectional within-person cross-lagged effects between depressive symptoms and cannabis consequences, while those below thresholds had no cross-lagged relationships, i.e., all cross-lagged effects appear to be driven by those above clinical thresholds at baseline. Similar sex differences and depression-threshold differences were found in the full sample (See SM for extended subgroup results).

**Table 3.** Stratified effects based on sex assigned at birth (Model 3; n = 517 female, n = 444 male)^a^

	Depressive Symptoms		Anxiety Symptoms	
	Consequences		Consequences	
	Female	Male	Female	Male
Within-Person: Cannabis to Internalizing				
T1 ➔ T2	**0.146 (0.04) p < 0.001**	−0.084 (0.06) p = 0.176	**0.129 (0.04) p = 0.001**	−0.98 (0.07) p = 0.146
T2 ➔ T3	**0.148 (0.04) p < 0.001**	−0.073 (0.05) p = 0.131	**0.116 (0.04) p = 0.002**	−0.083 (0.05) p = 0.115
T3 ➔ T4	**0.126 (0.04) p = 0.001**	−0.066 (0.04) p = 0.144	**0.110 (0.03) p = 0.001**	−0.078 (0.05) p = 0.127
T4 ➔ T5	**0.151 (0.04) p < 0.001**	−0.066 (0.04) p = 0.122	**0.136 (0.04) p = 0.001**	−0.079 (0.05) p = 0.111
T5 ➔ T6	**0.150 (0.04) p < 0.001**	−0.066 (0.05) p = 0.149	**0.116 (0.04) p = 0.001**	−0.073 (0.05) p = 0.134
T6 ➔ T7	**0.174 (0.05) p = 0.001**	−0.058 (0.04) p = 0.114	**0.140 (0.04) p = 0.001**	−0.069 (0.04) p = 0.113
Within-Person: Internalizing to Cannabis				
T1 ➔ T2	**0.072 (0.03) p = 0.020**	0.027 (0.04) p = 0.458	**0.055 (0.03) p = 0.045**	−0.026 (0.04) p = 0.526
T2 ➔ T3	**0.076 (0.04) p = 0.028**	0.027 (0.04) p = 0.456	0.058 (0.03) p = 0.052	−0.028 (0.04) p = 0.518
T3 ➔ T4	**0.066 (0.03) p = 0.031**	0.031 (0.04) p = 0.450	0.056 (0.03) p = 0.057	−0.033 (0.05) p = 0.522
T4 ➔ T5	**0.086 (0.04) p = 0.028**	0.032 (0.04) p = 0.450	0.065 (0.03) p = 0.052	−0.034 (0.05) p = 0.524
T5 ➔ T6	**0.071 (0.03) p = 0.021**	0.034 (0.04) p = 0.440	**0.052 (0.03) p = 0.048**	−0.034 (0.05) p = 0.527
T6 ➔ T7	**0.082 (0.04) p = 0.031**	0.030 (0.04) p = 0.464	0.070 (0.04) p = 0.053	−0.032 (0.05) p = 0.502
Within-Person: Cannabis to Cannabis				
T1➔ T2	**0.269 (0.06); p < 0.001**	0.026 (0.097); p = 0.785	**0.269 (0.06); p < 0.001**	0.043 (0.09); p = 0.651
T2 ➔ T3	**0.253 (0.06); p < 0.001**	0.028 (0.103); p = 0.788	**0.253 (0.06); p < 0.001**	0.044 (0.10); p = 0.659
T3 ➔ T4	**0.238 (0.06); p < 0.001**	0.027 (0.099); p = 0.789	**0.233 (0.06); p < 0.001**	0.043 (0.10); p = 0.663
T4 ➔ T5	**0.280 (0.07); p < 0.001**	0.026 (0.097); p = 0.790	**0.277 (0.07); p < 0.001**	0.042 (0.10); p = 0.665
T5 ➔ T6	**0.234 (0.06); p < 0.001**	0.028 (0.104); p = 0.786	**0.232 (0.06); p < 0.001**	0.044 (0.10); p = 0.654
T6 ➔ T7	**0.321 (0.07); p < 0.001**	0.022 (0.083); p = 0.789	**0.318 (0.07); p < 0.001**	0.037 (0.08); p = 0.661
Within-Person: Internalizing to Internalizing				
T1➔ T2	0.045 (0.05); p = 0.396	0.035 (0.060); p = 0.554	**0.115 (0.04); p = 0.006**	−0.026 (0.04); p = 0.526
T2 ➔ T3	0.051 (0.06); p = 0.418	0.029 (0.050); p = 0.558	**0.115 (0.05); p = 0.012**	−0.028 (0.04); p = 0.518
T3 ➔ T4	0.040 (0.05); p = 0.406	0.032 (0.055); p = 0.555	**0.114 (0.04); p = 0.010**	−0.033 (0.05); p = 0.522
T4 ➔ T5	0.053 (0.07); p = 0.412	0.034 (0.057); p = 0.554	**0.139 (0.05); p = 0.008**	−0.034 (0.05); p = 0.524
T5 ➔ T6	0.052 (0.06); p = 0.397	0.033 (0.055); p = 0.553	**0.113 (0.04); p = 0.007**	−0.034 (0.05); p = 0.527
T6 ➔ T7	0.051 (0.06); p = 0.417	0.032 (0.055); p = 0.561	**0.134 (0.05); p = 0.011**	−0.032 (0.05); p = 0.502
Between-Person: Cannabis Intercept with				
Internalizing Intercept	**0.337 (0.07) p < 0.001**	**0.299 (0.08) p < 0.001**	**0.259 (0.08) p = 0.001**	**0.341 (0.09) p < 0.001**
Cannabis Slope	**−0.858 (0.06) p < 0.001**	**−0.505 (0.10) p < 0.001**	**−0.851 (0.05) p < 0.001**	**−0.508 (0.10) p < 0.001**
Internalizing Slope	−0.178 (0.12) p = 0.145	−0.171 (0.12) p = 0.146	0.001 (0.14) p = 0.995	−0.232 (0.27) p = 0.398
Between-Person: Cannabis Slope with				
Internalizing Intercept	**−0.254 (0.11) p = 0.017**	−0.041 (0.12) p = 0.734	−0.201 (0.12) p = 0.082	−0.074 (0.14) p = 0.601
Internalizing Slope	0.218 (0.16) p = 0.185	0.345 (0.18) p = 0.057	0.088 (0.20) p = 0.657	0.419 (0.43) p = 0.335
Between-Person: Internalizing Intercept with				
Internalizing Slope	**−0.456 (0.09) p < 0.001**	**−0.303 (0.15) p = 0.047**	**−0.456 (0.09) p < 0.001**	0.085 (0.53) p = 0.857

a Adjusted for between-person effects of age, sex, race, sexual orientation, degree, trauma, and family history.

**Table 4.** Stratified effects based on clinical cut-offs at baseline (Model 3; PHQ: n = 324 above thresholds)^a^

	Cannabis Consequences & Depressive Symptoms	
	Sub-Clinical	Clinical
Within-Person: Cannabis to Internalizing		
T1 ➔ T2	0.002 (0.04) p > 0.9	**0.201 (0.06) p < 0.001**
T2 ➔ T3	0.002 (0.04) p > 0.9	**0.185 (0.06) p = 0.001**
T3 ➔ T4	0.001 (0.04) p > 0.9	**0.177 (0.06) p = 0.003**
T4 ➔ T5	0.001 (0.04) p > 0.9	**0.182 (0.06) p = 0.003**
T5 ➔ T6	0.001 (0.03) p > 0.9	**0.196 (0.06) p = 0.001**
T6 ➔ T7	0.001 (0.03) p > 0.9	**0.227 (0.07) p = 0.002**
Within-Person: Internalizing to Cannabis		
T1 ➔ T2	0.000 (0.02) p > 0.9	**0.091 (0.03) p = 0.004**
T2 ➔ T3	0.000 (0.03) p > 0.9	**0.114 (0.04) p = 0.008**
T3 ➔ T4	0.000 (0.03) p > 0.9	**0.127 (0.04) p = 0.005**
T4 ➔ T5	0.000 (0.04) p > 0.9	**0.137 (0.05) p = 0.007**
T5 ➔ T6	0.000 (0.04) p > 0.9	**0.113 (0.04) p = 0.004**
T6 ➔ T7	0.000 (0.03) p > 0.9	**0.117 (0.05) p = 0.013**
Within-Person: Cannabis to Cannabis		
T1 ➔ T2	0.123 (0.09); p = 0.166	**0.360 (0.08); p < 0.001**
T2 ➔ T3	0.112 (0.09); p = 0.197	**0.357 (0.07); p < 0.001**
T3 ➔ T4	0.118 (0.09); p = 0.172	**0.368 (0.10); p < 0.001**
T4 ➔ T5	0.138 (0.10); p = 0.171	**0.368 (0.09); p < 0.001**
T5 ➔ T6	0.113 (0.09); p = 0.202	**0.313 (0.08); p < 0.001**
T6 ➔ T7	0.104 (0.08); p = 0.164	**0.411 (0.10); p < 0.001**
Within-Person: Internalizing to Internalizing		
T1 ➔ T2	**0.055 (0.03); p = 0.040**	0.056 (0.06); p = 0.332
T2 ➔ T3	**0.095 (0.05); p = 0.037**	0.064 (0.07); p = 0.358
T3 ➔ T4	**0.081 (0.04); p = 0.037**	0.066 (0.07); p = 0.335
T4 ➔ T5	**0.101 (0.05); p = 0.032**	0.074 (0.08); p = 0.347
T5 ➔ T6	**0.088 (0.04); p = 0.038**	0.077 (0.08); p = 0.324
T6 ➔ T7	**0.099 (0.05); p = 0.035**	0.071 (0.08); p = 0.363
Between-Person: Cannabis Intercept with		
Internalizing Intercept	**0.183 (0.08) p = 0.024**	0.026 (0.16) p = 0.867
Cannabis Slope	**−0.683 (0.09) p < 0.001**	**−0.864 (0.27) p = 0.001**
Internalizing Slope	−0.035 (0.09) p = 0.708	0.230 (0.18) p = 0.211
Between-Person: Cannabis Slope with		
Internalizing Intercept	−0.090 (0.12) p = 0.465	0.367 (0.48) p = 0.447
Internalizing Slope	0.147 (0.15) p = 0.337	−0.332 (0.59) p = 0.575
Between-Person: Internalizing Intercept with		
Internalizing Slope	0.317 (0.22) p = 0.144	0.051 (0.30) p = 0.866

a Adjusted for between-person effects of age, sex, race, sexual orientation, degree, trauma, and family history.

## Discussion

This study examined bidirectional longitudinal relationships between cannabis (frequency and consequences) and internalizing symptoms (depressive and anxiety symptoms) among high-risk emerging adults. Consistent with prior research, both cannabis-related factors and internalizing symptoms decreased across emerging adulthood (Cristiano & Sharif-Razi, 2019; Lee & Sher, 2018). As hypothesized, higher cannabis frequency and consequences at baseline were associated with higher internalizing symptoms, aligning with research showing cannabis use consistently co-occurs with internalizing symptoms, and that this co-occurrence has been increasing among adults in recent years (Gorfinkel et al., 2020; Halladay, Munn, et al., 2019b; Han et al., 2021; Pacek et al., 2020). However, between-person (average overall) changes in cannabis frequency or consequences and internalizing variables over time did not appear to be related. Significant within-person bidirectional relationships were found, primarily between cannabis consequences and depressive symptoms. The presence and magnitude of these bidirectional relationships varied depending on the specific cannabis-related measure, type of internalizing problem, other substance use adjustments, and subgroups being explored.

Supporting the substance-induced hypothesis, higher within-person cannabis consequences predicted later increases in depressive and anxiety symptoms (Gobbi et al., 2019; Lev-Ran et al., 2014). This pathway was typically found for cannabis consequences, rather than cannabis frequency. This contrasts with previous work among emerging adults that has found links between cannabis frequency and internalizing symptoms, though previously explored time-lags were year(s) in duration rather than months (Bolanis et al., 2020; Davis et al., 2022; Davis et al., 2023a, 2023b; Womack et al., 2016). Rather, in the current sample, using 4-month waves, substance-induced pathways may be driven by psychosocial mechanisms (e.g., interpersonal, academic/occupational, risky behaviors) or non-frequency-related symptoms of cannabis use disorder (e.g., blackout, impaired control, physiological dependence). However, potency and quantity of use per day were not measured, making this conjectural.

Providing partial support for the symptom-driven hypothesis, depressive symptoms predicted subsequent increases in cannabis consequences, though these pathways were sensitive to covariate adjustments. This pattern is consistent with prior research demonstrating significant temporal associations from internalizing symptoms – especially depression – to cannabis use (Amendola et al., 2022; Davis et al., 2023b; London-Nadeau et al., 2021; Rhew et al., 2017). Using substances to cope with symptoms is the typical explanation for symptom-driven pathways (Moitra et al., 2015). The lack of directional relationships for cannabis frequency in the current study may suggest that depressive symptoms change the context and consequences of cannabis use, rather than its’ frequency, or may be due to limited frequency options (i.e., no options between weekly and daily or differentiation between using once per day versus multiple times per day). In summary, bidirectional pathways were largely found between depressive symptoms and cannabis consequences, while unidirectional substance-induced pathways emerged for anxiety symptoms and cannabis consequences. No consistent directional pathways were found for cannabis frequency.

Effects suggestive of symptom-driven pathways appeared more sensitive to time-varying covariate adjustments, particularly alcohol, than effects suggestive of substance-induced pathways. In the case of depressive symptoms, accounting for time-varying drinks per week revealed new associations with later increases in cannabis frequency, although the link to subsequent consequences was no longer significant. For anxiety, predictive associations with later cannabis consequences emerged when adjusting for time-varying cigarette use or income but remained non-significant when alcohol was accounted for. Although some prior studies accounted for other substance use at baseline, many did not, and few incorporated time-varying changes. Of those including adjustments, two prior studies similarly found stronger connections between increases in cannabis variables preceding increases in internalizing symptoms, rather than the reverse, after adjusting for other substance use (Duperrouzel et al., 2018; London-Nadeau et al., 2021), though another study reported the opposite pattern – where controlling for other substance use nullified cannabis-to-internalizing symptom effects, while internalizing-to-cannabis variable effects remained significant (Bolanis, 2020). These nuanced findings underscore the importance of thoughtful adjustments of other substance use or direct modeling of patterns of multiple use in future research. This includes exploring how general polysubstance use (i.e., use of more than one substance, regardless of whether they are used simultaneously or separately) and co-use (i.e., concurrent or simultaneous use of multiple substances) impacts these pathways, both of which have previously been associated with more negative psychosocial and mental health outcomes relative to using only one substances (Halladay et al., 2020; Karoly, Ross, Ellingson, & Feldstein-Ewing, 2020; Yurasek, Aston, & Metrik, 2017).

Across models, relationships were generally stronger and more consistent for depressive symptoms and cannabis consequences. Stronger directional relationships with depressive symptoms compared to anxiety is supported by prior work (Amendola et al., 2022; Colder et al., 2019; Duperrouzel et al., 2018; Gobbi et al., 2019; London-Nadeau et al., 2021) and highlights the importance of examining depression and anxiety separately, rather than as a combined internalizing factor. Greater consistency with cannabis consequences, rather than frequency, may suggest that: (1) cannabis consequences are, in part, manifestations of negative affect (i.e., experiencing conflict with others, regretting outcomes, feeling guilty about use), and/or (2) experiencing significant cannabis-related consequences may trigger increases in depressive and anxiety symptoms (i.e., feeling sad or anxious following adverse cannabis-related health or social consequences), and/or (3) there are other (not measured) stressors, health, or sociocontextual factors that influence both cannabis consequences and internalizing symptoms, but not cannabis frequency. Notably, when cannabis-attributable internalizing consequences were excluded from analyses, links remained between other cannabis consequences predicting later anxiety symptoms and depressive symptoms predicting subsequent cannabis consequences. Thus, it is not only the internalizing specific consequences that drive relationships between cannabis consequences and heightened internalizing symptoms. This supports using consequence-based measures for screening and brief interventions that are focused on reducing consequences, rather than just reducing use, and integrating mood and emotional regulation strategies (Martínez-Vispo, Martínez, López-Durán, del Rio, & Becoña, 2018; Murphy et al., 2024).

Stronger bidirectional relationships found between cannabis consequences and internalizing symptoms among females compared to males is supported by prior work (Davis et al., 2022; Halladay, Boyle, et al., 2019a). Cannabis may impact females differently due to sex differences in hormones, pharmacokinetics, and the endocannabinoid system (Bassir Nia et al., 2018; Greaves & Hemsing, 2020). This may be connected to the present findings, as cannabis consequences are partly determined by physiological dependence (Simons et al., 2012). Sociocultural experiences related to gender roles, identity, and marginalization may also contribute to stronger relationships in women (Greaves & Hemsing, 2020). Broader gender disparities related to opportunities, resources, behavioral expectations, motivations, power, and relationships are connected to various subdomains of cannabis consequences (e.g., interpersonal, academic/occupational, self-perception domains; Simons et al., 2012) and may contribute to differential motives for use. It is important to note that similar sex-specific relationships for cannabis frequency were not found, and these results were based on simplified models. Future work is needed to disentangle specific sex- and/or gender-related mechanisms and differential pathways to co-occurrence.

Stronger bidirectional relationships with cannabis consequences were also found for emerging adults who began the study with elevated depressive symptoms that surpassed established clinical cut-offs. A previous study similarly found that in emerging adults with elevated depressive symptoms, higher cannabis frequency predicted later increases in symptoms, a relationship not found among those below symptom thresholds (Womack et al., 2016). This is consistent with clinical research findings showing that cannabis frequency is high among young people with clinical levels of internalizing symptoms (Halladay et al., 2021; Heradstveit, Skogen, Hetland, Stewart, & Hysing, 2019) and that reductions in cannabis frequency are associated with reductions in clinical internalizing symptoms (Hser et al., 2017; Moitra, Anderson, & Stein, 2016). As such, development and refinement of targeted prevention and early intervention efforts are merited for emerging adults experiencing elevated depressive symptoms, such as tailoring cannabis brief interventions for young people accessing care for mental health concerns or surpassing clinical symptom thresholds (Halladay et al., 2019c; Halladay, Fein, MacKillop, & Munn, 2018). Treatment approaches that also address internalizing symptoms, by addressing coping skills, relaxation, and substance-free activities, may be especially effective (Côté et al., 2024; Halladay, Scherer, et al., 2019c; Murphy et al., 2024; Stephens et al., 2020).

This study has several strengths, including leveraging contemporary statistical approaches to explore bidirectional between- and within-person relationships across 2 years of emerging adulthood in a large and diverse high-risk sample. However, limitations need to be considered when interpreting findings. First, both cannabis and symptom measures use self-reported scales rather than objective or diagnostic assessments; notably, assessments used are common measures and have been validated in similar samples (Byrd-Bredbenner, Eck, & Quick, 2021; Moriarty, Gilbody, McMillan, & Manea, 2015; Read, Egerton, Cheesman, & Steers, 2022). Moreover, our cannabis use measure did not assess quantity of use, product type, or potency, which may have limited our ability to detect prospective associations with internalizing symptoms. Second, it is likely that directional relationships would differ if exploring different developmental ages, longer periods of observation, or using shorter intervals between waves. For example, recent work exploring these relationships on the day-to-day level has found mostly null relationships (Dora et al., 2023; Litt et al., 2023). Third, the sample represented emerging adults engaging in relatively frequent drinking and cannabis use; directional relationships may differ in other samples. Future work is thus needed to identify and understand these subgroup differences. Last, despite controlling for many potential confounders, as with all observational studies, there is a risk of residual confounding due to unmeasured confounders (e.g., externalizing problems, sociocultural stressors). The current study also faced model convergence issues, prohibiting comprehensive time-varying covariate adjustments across all main and sub-group models, limiting the ability to isolate cannabis from other substance-related pathways.

Overall, this study highlights the nuanced bidirectional relationships between cannabis related variables and internalizing symptoms across emerging adulthood, with meaningful variation based on the type of internalizing symptoms and related clinical severity, type of cannabis measurement, other substance use adjustments, and sex assigned at birth. These findings underscore the importance of tailored prevention and early intervention messaging, including: (1) taking a harm reduction lens to reduce cannabis consequences, rather than focusing solely on frequency of use; (2) developing targeted strategies for emerging adults with clinically elevated internalizing symptoms; and (3) prioritizing early identification and supports for females.

## Supporting information

Halladay et al. supplementary material 1 Halladay et al. supplementary material

Halladay et al. supplementary material 2 Halladay et al. supplementary material

## Footnotes

1 Cannabis frequency and anxiety models including total drinks per week as a time-varying confounder did not converge (even with constraining residual variances and removing alcohol slopes). This is unlikely to be due to collinearity between cannabis frequency and drinks per week (r = 0.05 and 0.2, respectively).

2 While not in the pre-registration, attempts were made to run time-varying adjusted stratified models. These did not converge with non-ignorable and non-resolvable Heywood cases.

## Supplementary material

The supplementary material for this article can be found at http://doi.org/10.1017/S0033291725101700.
