# Supplementary material for: Examining bidirectional associations between cannabis use and internalizing symptoms among high-risk emerging adults: A prospective cohort study
Source: Psychol Med. 2025 Oct 3;55:e291. doi: 10.1017/S0033291725101700 (PMC12527518; doi:10.1017/S0033291725101700)
Supplement: Halladay et al. supplementary material 1 — Halladay et al. supplementary material [file S0033291725101700sup001.docx]

**Examining bidirectional associations between cannabis use and internalizing symptoms among high-risk emerging adults: A prospective cohort study**

**SUPPLEMENTARY MATERIALS**

1. Model fit

Linear models were chosen as best function of time because the quadratic term and slope terms were highly collinear (> 0.8 for all except depression which was still high (0.76), and did not have significant slope or quadratic terms).

Table S1. Linear Growth Curves to determine function of time.

| **Measure** | **Growth** | **AIC** | **BIC** | **SA-BIC** | **CFI** | **TLI** | **RMSEA** |
| --- | --- | --- | --- | --- | --- | --- | --- |
| Anxiety | No Growth | 29927.371 | 29971.173 | 29942.59 | 0.93 | 0.944 | 0.065 (0.054 - 0.076) |
| **Anxiety** | **Linear Growth** | **29830.237** | **29888.64** | **29850.529** | **0.979** | **0.981** | **0.038 (0.026 - 0.051)** |
| Anxiety | Quadratic growth | 29815.417 | 29893.288 | 29842.472 | 0.987 | 0.986 | 0.033 (0.017 - 0.047) |
| Depression | No Growth | 30631.851 | 30675.653 | 30647.07 | 0.932 | 0.945 | 0.062 (0.051 - 0.073) |
| Depression | **Linear Growth** | **30515.923** | **30574.326** | **30536.214** | **0.99** | **0.991** | **0.025 (0.006 - 0.039)** |
| Depression | Quadratic growth | 30509.985 | 30587.856 | 30537.04 | 0.994 | 0.994 | 0.021 (0 - 0.038) |
| Cannabis Frequency | No Growth | - | - | - | 0.962 | 0.979 | 0.1 (0.092 - 0.109) |
| **Cannabis Frequency** | **Linear Growth** | - | - | - | **0.994** | **0.996** | **0.041 (0.031 - 0.051)** |
| Cannabis Frequency | Quadratic growth | - | - | - | 0.999 | 0.999 | 0.02 (0 - 0.034) |
| Cannabis Consequences | No Growth | 25862.066 | 25905.869 | 25877.285 | 0.847 | 0.877 | 0.076 (0.065 - 0.087) |
| Cannabis Consequences | Linear Growth | **25557.101** | **25615.504** | **25577.392** | **0.974** | **0.976** | **0.033 (0.02 - 0.047)** |
| Cannabis Consequences | Quadratic growth | 25484.25 | 25562.121 | 25511.306 | 1 | 1 | 0 (0 - 0.024) |

Table S2. RI-CLPM model fit to determine constraints of the lagged effects.

| **Variables** | **Lagged Constraints** | **AIC** | **BIC** | **SA.BIC** | **CFI** | **TLI** | **ChiSq** | **ChiSqDF** | **ChiSqSCF** | **RMSEA** |
| --- | --- | --- | --- | --- | --- | --- | --- | --- | --- | --- |
| Cannabis Frequency & Anxiety | Unconstrained | - | - | - | 0.99 | 0.984 | 134.904 | 57 | - | 0.038 (0.03 - 0.046) |
| Cannabis Frequency & Anxiety | Auto-Lagged Constrained | - | - | - | 0.991 | 0.988 | 137.281 | 67 | - | 0.033 (0.025 - 0.041) |
| Cannabis Frequency & Anxiety | **Full (Auto- and Cross-Lagged) Constrained** | - | - | - | **0.992** | **0.991** | **136.877** | **77** | **-** | **0.028 (0.021 - 0.036)** |
| Cannabis Frequency & Depression | Unconstrained | - | - | - | 0.991 | 0.986 | 126.221 | 57 | - | 0.036 (0.027 - 0.044) |
| Cannabis Frequency & Depression | Auto-Lagged Constrained | - | - | - | 0.991 | 0.988 | 136.305 | 67 | - | 0.033 (0.025 - 0.041) |
| Cannabis Frequency & Depression | **Full (Auto- and Cross-Lagged) Constrained** | - | - | - | **0.992** | **0.99** | **143.912** | **77** | **-** | **0.03 (0.022 - 0.038)** |
| Cannabis Consequences & Anxiety | Unconstrained | 55174.845 | 55476.595 | 55279.685 | 0.987 | 0.979 | 98.683 | 57 | 1.5601 | 0.028 (0.018 - 0.037) |
| Cannabis Consequences & Anxiety | Auto-Lagged Constrained | 55184.757 | 55437.838 | 55272.687 | 0.988 | 0.983 | 106.108 | 67 | 1.7328 | 0.025 (0.015 - 0.033) |
| Cannabis Consequences & Anxiety | **Full (Auto- and Cross-Lagged) Constrained** | **55183.682** | **55388.093** | **55254.702** | **0.987** | **0.984** | **119.374** | **77** | **1.6988** | **0.024 (0.015 - 0.032)** |
| Cannabis Consequences & Depression | Unconstrained | 55835.223 | 56136.973 | 55940.063 | 0.983 | 0.973 | 111.143 | 57 | 1.581 | 0.031 (0.023 - 0.04) |
| Cannabis Consequences & Depression | Auto-Lagged Constrained | 55861.252 | 56114.332 | 55949.182 | 0.981 | 0.974 | 127.508 | 67 | 1.739 | 0.031 (0.022 - 0.039) |
| Cannabis Consequences & Depression | **Full (Auto- and Cross-Lagged) Constrained** | **55859.723** | **56064.135** | **55930.744** | **0.98** | **0.977** | **139.377** | **77** | **1.7235** | **0.029 (0.021 - 0.037)** |

1. Extended Descriptive Statistics

Table S3. Date range and number of participants with observations at a given period.

| Period | P1 | P2 | P3 | P4 | P5 | P6 | P7 |
| --- | --- | --- | --- | --- | --- | --- | --- |
| Approx. Date | up to March 2018 | March – July 2018 | July – Nov 2018 | Nov 2018 – March 2019 | March – July 2019 | July to Nov 2019 | Nov 2019 – March 2020 |
| N | 602 | 759 | 759 | 764 | 769 | 725 | 702 |

Table S4. Descriptive characteristics by site.

| **Characteristic** | **Overall Sample**  **N = 961** | **Hamilton Sample**  **N = 574** | **Memphis Sample**  **N = 387** | **p-value** |
| --- | --- | --- | --- | --- |
| Age | 21.89 +/- 1.25 | 21.41 +/- 1.17 | 22.59 +/- 1.02 | <0.001 |
| Sex Assigned at Birth |  |  |  | 0.3 |
| Female | 517 (53.80%) | 301 (52.44%) | 216 (55.81%) | |
| Male | 444 (46.20%) | 273 (47.56%) | 171 (44.19%) | |
| Race | |  |  | <0.001 |
| Black | 180 (18.73%) | 14 (2.44%) | 166 (42.89%) | |
| Asian | 110 (11.45%) | 103 (17.94%) | 7 (1.81%) |  |
| Other | 99 (10.30%) | 55 (9.58%) | 44 (11.37%) | |
| White | 572 (59.52%) | 402 (70.03%) | 170 (43.93%) | |
| Sexual Orientation | |  |  | 0.7 |
| Heterosexual | 686 (71.38%) | 413 (71.95%) | 273 (70.54%) | |
| 2SLGBTQIA+ | 275 (28.62%) | 161 (28.05%) | 114 (29.46%) | |
| Subjective Household Income |  |  |  | <0.001 |
| Not Enough | 49 (5.10%) | 22 (3.83%) | 27 (6.98%) | |
| Cut Back | 269 (27.99%) | 131 (22.82%) | 138 (35.66%) | |
| No Extras | 301 (31.32%) | 193 (33.62%) | 108 (27.91%) | |
| Enough for Extras | 342 (35.59%) | 228 (39.72%) | 114 (29.46%) | |
| 4-Year Educational Outcome | |  |  | >0.9 |
| Less than a Bachelors | 127 (13.22%) | 77 (13.41%) | 50 (12.92%) | |
| Bachelors or Higher | 834 (86.78%) | 497 (86.59%) | 337 (87.08%) | |
| Cannabis ASSIST (Frequency) | |  |  | <0.001 |
| None | 195 (20.31%) | 123 (21.43%) | 72 (18.65%) | |
| Monthly | 353 (36.77%) | 228 (39.72%) | 125 (32.38%) | |
| Week | 184 (19.17%) | 111 (19.34%) | 73 (18.91%) | |
| Daily | 103 (10.73%) | 60 (10.45%) | 43 (11.14%) | |
| Daily+ | 125 (13.02%) | 52 (9.06%) | 73 (18.91%) | |
| Missing | 1 | 0 | 1 |  |
| MACQ-Brief (21-Item) Score | 3.33 +/- 4.19 | 3.18 +/- 4.29 | 3.56 +/- 4.03 | 0.2 |
| Missing | 4 | 2 | 2 |  |
| CUDIT Score | 7.03 +/- 6.74 | 6.58 +/- 6.65 | 7.70 +/- 6.83 | 0.011 |
| Missing | 2 | 1 | 1 |  |
| PHQ-9 Score | 7.98 +/- 5.81 | 8.09 +/- 5.91 | 7.80 +/- 5.66 | 0.4 |
| Missing | 3 | 1 | 2 |  |
| GAD-7 Score | 6.49 +/- 5.42 | 6.64 +/- 5.53 | 6.28 +/- 5.25 | 0.3 |
| Missing | 4 | 2 | 2 |  |

Table S5. Descriptive characteristics by GAD7- Symptoms.

| **Characteristic** | **Overall**  **N = 957** | **Sub-Clinical**  **N = 717** | **Clinical**  **N = 240** | **p-value** |
| --- | --- | --- | --- | --- |
| Sample |  |  |  | 0.4 |
| Hamilton | 572 (59.77%) | 423 (59.00%) | 149 (62.08%) | |
| Memphis | 385 (40.23%) | 294 (41.00%) | 91 (37.92%) | |
| Age | 21.89 +/- 1.25 | 21.93 +/- 1.22 | 21.74 +/- 1.35 | 0.039 |
| Sex Assigned at Birth |  |  |  | <0.001 |
| Female | 514 (53.71%) | 355 (49.51%) | 159 (66.25%) | |
| Male | 443 (46.29%) | 362 (50.49%) | 81 (33.75%) | |
| Race | |  |  | 0.10 |
| Black | 179 (18.70%) | 133 (18.55%) | 46 (19.17%) | |
| Asian | 110 (11.49%) | 91 (12.69%) | 19 (7.92%) | |
| Other | 99 (10.34%) | 79 (11.02%) | 20 (8.33%) | |
| White | 569 (59.46%) | 414 (57.74%) | 155 (64.58%) | |
| Sexual Orientation | |  |  | <0.001 |
| Heterosexual | 683 (71.37%) | 548 (76.43%) | 135 (56.25%) | |
| 2SLGBTQIA+ | 274 (28.63%) | 169 (23.57%) | 105 (43.75%) | |
| Subjective Household Income |  |  |  | <0.001 |
| Not Enough | 49 (5.12%) | 30 (4.18%) | 19 (7.92%) | |
| Cut Back | 267 (27.90%) | 180 (25.10%) | 87 (36.25%) | |
| No Extras | 300 (31.35%) | 224 (31.24%) | 76 (31.67%) | |
| Enough for Extras | 341 (35.63%) | 283 (39.47%) | 58 (24.17%) | |
| 4-Year Educational Outcome | |  |  | <0.001 |
| Less than a Bachelors | 127 (13.27%) | 77 (10.74%) | 50 (20.83%) | |
| Bachelors or Higher | 830 (86.73%) | 640 (89.26%) | 190 (79.17%) | |
| Cannabis ASSIST (Frequency) | |  |  | <0.001 |
| None | 195 (20.40%) | 159 (22.21%) | 36 (15.00%) | |
| Monthly | 352 (36.82%) | 276 (38.55%) | 76 (31.67%) | |
| Week | 184 (19.25%) | 131 (18.30%) | 53 (22.08%) | |
| Daily | 102 (10.67%) | 75 (10.47%) | 27 (11.25%) | |
| Daily+ | 123 (12.87%) | 75 (10.47%) | 48 (20.00%) | |
| Missing | 1 | 1 | 0 |  |
| MACQ-Brief (21-Item) Score | 3.33 +/- 4.19 | 2.87 +/- 3.78 | 4.69 +/- 5.01 | <0.001 |
| Missing | 1 | 1 | 0 |  |
| CUDIT Score | 7.00 +/- 6.72 | 6.25 +/- 6.09 | 9.25 +/- 7.92 | <0.001 |
| Missing | 2 | 2 | 0 |  |
| PHQ-9 Score | 7.98 +/- 5.81 | 5.85 +/- 4.16 | 14.36 +/- 5.35 | <0.001 |
| GAD-7 Score | 6.49 +/- 5.42 | 3.86 +/- 2.70 | 14.37 +/- 3.53 | <0.001 |

Table S6. Descriptive characteristics by PHQ-9 Symptoms.

| **Characteristic** | **Overall**  **N = 958** | **Sub-Clinical**  **N = 634** | **Clinical**  **N = 324** | **p-value** |
| --- | --- | --- | --- | --- |
| Sample |  |  |  | >0.9 |
| Hamilton | 573 (59.81%) | 380 (59.94%) | 193 (59.57%) | |
| Memphis | 385 (40.19%) | 254 (40.06%) | 131 (40.43%) | |
| Age | 21.89 +/- 1.25 | 21.93 +/- 1.21 | 21.80 +/- 1.33 | 0.12 |
| Sex Assigned at Birth |  |  |  | <0.001 |
| Female | 515 (53.76%) | 316 (49.84%) | 199 (61.42%) | |
| Male | 443 (46.24%) | 318 (50.16%) | 125 (38.58%) | |
| Race | |  |  | 0.4 |
| Black | 179 (18.68%) | 114 (17.98%) | 65 (20.06%) | |
| Asian | 110 (11.48%) | 80 (12.62%) | 30 (9.26%) | |
| Other | 99 (10.33%) | 67 (10.57%) | 32 (9.88%) | |
| White | 570 (59.50%) | 373 (58.83%) | 197 (60.80%) | |
| Sexual Orientation | |  |  | <0.001 |
| Heterosexual | 684 (71.40%) | 493 (77.76%) | 191 (58.95%) | |
| 2SLGBTQIA+ | 274 (28.60%) | 141 (22.24%) | 133 (41.05%) | |
| Subjective Household Income |  |  |  | <0.001 |
| Not Enough | 49 (5.11%) | 19 (3.00%) | 30 (9.26%) | |
| Cut Back | 267 (27.87%) | 155 (24.45%) | 112 (34.57%) | |
| No Extras | 300 (31.32%) | 198 (31.23%) | 102 (31.48%) | |
| Enough for Extras | 342 (35.70%) | 262 (41.32%) | 80 (24.69%) | |
| 4-Year Educational Outcome | |  |  | <0.001 |
| Less than a Bachelors | 127 (13.26%) | 67 (10.57%) | 60 (18.52%) | |
| Bachelors or Higher | 831 (86.74%) | 567 (89.43%) | 264 (81.48%) | |
| Cannabis ASSIST (Frequency) | |  |  | <0.001 |
| None | 195 (20.38%) | 148 (23.34%) | 47 (14.55%) | |
| Monthly | 353 (36.89%) | 243 (38.33%) | 110 (34.06%) | |
| Week | 184 (19.23%) | 122 (19.24%) | 62 (19.20%) | |
| Daily | 102 (10.66%) | 63 (9.94%) | 39 (12.07%) | |
| Daily+ | 123 (12.85%) | 58 (9.15%) | 65 (20.12%) | |
| Missing | 1 | 0 | 1 |  |
| MACQ-Brief (21-Item) Score | 3.33 +/- 4.19 | 2.69 +/- 3.69 | 4.59 +/- 4.79 | <0.001 |
| Missing | 2 | 1 | 1 |  |
| CUDIT Total | 7.00 +/- 6.72 | 5.93 +/- 5.86 | 9.09 +/- 7.75 | <0.001 |
| Missing | 2 | 0 | 2 |  |
| PHQ-9 Score | 7.98 +/- 5.81 | 4.59 +/- 2.82 | 14.60 +/- 4.25 | <0.001 |
| GAD-7 Score | 6.49 +/- 5.42 | 3.87 +/- 3.28 | 11.62 +/- 5.11 | <0.001 |
| Missing | 1 | 1 | 0 |  |

1. **Extended Main Model Results (Models 1-4)**

Table S7. Standardized estimates (unadjusted; Model 1)

|  | Depressive Symptoms | | Anxiety Symptoms | |
| --- | --- | --- | --- | --- |
|  | Frequency | Consequences | Frequency | Consequences |
| Within-Person: Cannabis to Internalizing | | | | |
| T1 🡪 T2 | -0.02 (0.04) p = 0.706 | 0.09 (0.04) p = 0.021 | 0.03 (0.05) p = 0.453 | 0.08 (0.04) p = 0.047 |
| T2 🡪 T3 | -0.01 (0.04) p = 0.707 | 0.08 (0.04) p = 0.022 | 0.03 (0.04) p = 0.450 | 0.06 (0.03) p = 0.047 |
| T3 🡪 T4 | -0.01 (0.04) p = 0.707 | 0.07 (0.03) p = 0.020 | 0.03 (0.04) p = 0.447 | 0.06 (0.03) p = 0.042 |
| T4 🡪 T5 | -0.01 (0.04) p = 0.707 | 0.08 (0.03) p = 0.024 | 0.03 (0.04) p = 0.450 | 0.07 (0.03) p = 0.045 |
| T5 🡪 T6 | -0.01 (0.04) p = 0.707 | 0.07 (0.03) p = 0.022 | 0.03 (0.04) p = 0.452 | 0.06 (0.03) p = 0.042 |
| T6 🡪 T7 | -0.02 (0.04) p = 0.707 | 0.08 (0.04) p = 0.028 | 0.03 (0.04) p = 0.451 | 0.07 (0.03) p = 0.048 |
| Within-Person: Internalizing to Cannabis | | | | |
| T1 🡪 T2 | 0.00 (0.04) p = 0.983 | 0.05 (0.03) p = 0.047 | 0.02 (0.04) p = 0.503 | 0.03 (0.02) p = 0.232 |
| T2 🡪 T3 | 0.00 (0.04) p = 0.983 | 0.05 (0.03) p = 0.050 | 0.02 (0.03) p = 0.503 | 0.03 (0.03) p = 0.239 |
| T3 🡪 T4 | 0.00 (0.04) p = 0.983 | 0.05 (0.03) p = 0.050 | 0.03 (0.04) p = 0.505 | 0.03 (0.03) p = 0.238 |
| T4 🡪 T5 | 0.00 (0.04) p = 0.983 | 0.06 (0.03) p = 0.049 | 0.03 (0.04) p = 0.506 | 0.04 (0.03) p = 0.236 |
| T5 🡪 T6 | 0.00 (0.04) p = 0.983 | 0.06 (0.03) p = 0.040 | 0.03 (0.04) p = 0.506 | 0.03 (0.03) p = 0.231 |
| T6 🡪 T7 | 0.00 (0.04) p = 0.983 | 0.05 (0.03) p = 0.052 | 0.03 (0.04) p = 0.504 | 0.03 (0.03) p = 0.246 |
| Between-Person: Cannabis Intercept with | | | | |
| Internalizing Intercept | 0.25 (0.05) p < 0.001 | 0.30 (0.05) p < 0.001 | 0.22 (0.06) p < 0.001 | 0.25 (0.05) p < 0.001 |
| Cannabis Slope | -0.43 (0.07) p < 0.001 | -0.62 (0.09) p < 0.001 | -0.43 (0.07) p < 0.001 | -0.62 (0.08) p < 0.001 |
| Internalizing Slope | -0.13 (0.09) p = 0.141 | -0.07 (0.08) p = 0.388 | -0.07 (0.11) p = 0.507 | 0.03 (0.10) p = 0.771 |
| Between-Person: Cannabis Slope with | | | | |
| Internalizing Intercept | -0.12 (0.07) p = 0.118 | -0.11 (0.10) p = 0.271 | -0.10 (0.07) p = 0.170 | -0.08 (0.11) p = 0.456 |
| Internalizing Slope | 0.23 (0.14) p = 0.091 | 0.13 (0.17) p = 0.461 | 0.15 (0.16) p = 0.354 | -0.01 (0.22) p = 0.978 |
| Between-Person: Internalizing Intercept with | | | | |
| Internalizing Slope | -0.44 (0.06) p < 0.001 | -0.45 (0.06) p < 0.001 | -0.43 (0.07) p < 0.001 | -0.42 (0.07) p < 0.001 |

Table S8. Standardized estimates (demographic-adjusted; Model 2).

|  | Depressive Symptoms | | Anxiety Symptoms | |
| --- | --- | --- | --- | --- |
|  | Frequency | Consequences | Frequency | Consequences |
| Within-Person: Cannabis to Internalizing | | | | |
| T1 🡪 T2 | -0.02 (0.04) p = 0.630 | 0.09 (0.04) p = 0.022 | 0.03 (0.04) p = 0.463 | 0.07 (0.04) p = 0.056 |
| T2 🡪 T3 | -0.02 (0.04) p = 0.631 | 0.08 (0.04) p = 0.024 | 0.03 (0.04) p = 0.463 | 0.06 (0.03) p = 0.057 |
| T3 🡪 T4 | -0.02 (0.04) p = 0.632 | 0.07 (0.03) p = 0.022 | 0.03 (0.04) p = 0.462 | 0.06 (0.03) p = 0.051 |
| T4 🡪 T5 | -0.02 (0.04) p = 0.631 | 0.08 (0.04) p = 0.026 | 0.03 (0.04) p = 0.465 | 0.06 (0.03) p = 0.054 |
| T5 🡪 T6 | -0.02 (0.04) p = 0.631 | 0.07 (0.03) p = 0.023 | 0.03 (0.04) p = 0.464 | 0.06 (0.03) p = 0.050 |
| T6 🡪 T7 | -0.02 (0.04) p = 0.631 | 0.08 (0.04) p = 0.030 | 0.03 (0.04) p = 0.463 | 0.06 (0.03) p = 0.058 |
| Within-Person: Internalizing to Cannabis | | | | |
| T1 🡪 T2 | -0.01 (0.03) p = 0.681 | 0.05 (0.03) p = 0.049 | 0.01 (0.03) p = 0.872 | 0.03 (0.02) p = 0.252 |
| T2 🡪 T3 | -0.01 (0.03) p = 0.681 | 0.05 (0.03) p = 0.052 | 0.00 (0.03) p = 0.872 | 0.03 (0.03) p = 0.259 |
| T3 🡪 T4 | -0.01 (0.04) p = 0.681 | 0.05 (0.03) p = 0.052 | 0.01 (0.04) p = 0.872 | 0.03 (0.03) p = 0.258 |
| T4 🡪 T5 | -0.02 (0.04) p = 0.681 | 0.06 (0.03) p = 0.051 | 0.01 (0.04) p = 0.872 | 0.03 (0.03) p = 0.256 |
| T5 🡪 T6 | -0.01 (0.04) p = 0.681 | 0.05 (0.03) p = 0.042 | 0.01 (0.04) p = 0.872 | 0.03 (0.03) p = 0.251 |
| T6 🡪 T7 | -0.01 (0.03) p = 0.681 | 0.05 (0.03) p = 0.055 | 0.01 (0.04) p = 0.872 | 0.03 (0.03) p = 0.266 |
| Between-Person: Cannabis Intercept with | | | | |
| Internalizing Intercept | 0.27 (0.06) p < 0.001 | 0.33 (0.05) p < 0.001 | 0.23 (0.06) p < 0.001 | 0.29 (0.06) p < 0.001 |
| Cannabis Slope | -0.45 (0.06) p < 0.001 | -0.65 (0.08) p < 0.001 | -0.45 (0.06) p < 0.001 | -0.65 (0.08) p < 0.001 |
| Internalizing Slope | -0.15 (0.09) p = 0.099 | -0.09 (0.08) p = 0.313 | -0.06 (0.11) p = 0.616 | 0.03 (0.10) p = 0.746 |
| Between-Person: Cannabis Slope with | | | | |
| Internalizing Intercept | -0.17 (0.08) p = 0.030 | -0.17 (0.10) p = 0.097 | -0.15 (0.08) p = 0.057 | -0.16 (0.11) p = 0.159 |
| Internalizing Slope | 0.25 (0.14) p = 0.067 | 0.14 (0.18) p = 0.429 | 0.14 (0.16) p = 0.368 | 0.03 (0.23) p = 0.911 |
| Between-Person: Internalizing Intercept with | | | | |
| Internalizing Slope | -0.40 (0.06) p < 0.001 | -0.41 (0.07) p < 0.001 | -0.36 (0.08) p < 0.001 | -0.36 (0.08) p < 0.001 |

Table S9. Time-varying models (fully adjusted; Model 4) with Drinks per week with exact p-values.

|  | Depressive Symptoms | | Anxiety Symptoms | |
| --- | --- | --- | --- | --- |
|  | Frequency | Consequences | Frequency | Consequences |
| Within-Person: Cannabis to Internalizing | | | | |
| T1 🡪 T2 | **0.16 (0.03) p < 0.001** | **0.09 (0.04) p = 0.023** | - | 0.07 (0.04) p = 0.061 |
| T2 🡪 T3 | **0.19 (0.04) p < 0.001** | **0.08 (0.04) p = 0.025** | - | 0.06 (0.03) p = 0.061 |
| T3 🡪 T4 | **0.17 (0.04) p < 0.001** | **0.07 (0.03) p = 0.024** | - | 0.06 (0.03) p = 0.056 |
| T4 🡪 T5 | **0.19 (0.04) p < 0.001** | **0.08 (0.04) p = 0.027** | - | 0.06 (0.03) p = 0.058 |
| T5 🡪 T6 | **0.19 (0.04) p < 0.001** | **0.08 (0.03) p = 0.025** | - | 0.06 (0.03) p = 0.055 |
| T6 🡪 T7 | **0.19 (0.04) p < 0.001** | **0.08 (0.04) p = 0.032** | - | 0.06 (0.03) p = 0.062 |
| Within-Person: Internalizing to Cannabis | | | | |
| T1 🡪 T2 | **0.13 (0.03) p < 0.001** | 0.05 (0.03) p = 0.052 | - | 0.03 (0.02) p = 0.293 |
| T2 🡪 T3 | **0.13 (0.03) p < 0.001** | 0.05 (0.03) p = 0.056 | - | 0.03 (0.03) p = 0.299 |
| T3 🡪 T4 | **0.13 (0.03) p < 0.001** | 0.05 (0.03) p = 0.055 | - | 0.03 (0.03) p = 0.299 |
| T4 🡪 T5 | **0.15 (0.03) p < 0.001** | 0.06 (0.03) p = 0.054 | - | 0.03 (0.03) p = 0.297 |
| T5 🡪 T6 | **0.14 (0.03) p < 0.001** | **0.05 (0.03) p = 0.045** | - | 0.03 (0.03) p = 0.292 |
| T6 🡪 T7 | **0.13 (0.03) p < 0.001** | 0.06 (0.03) p = 0.058 | - | 0.03 (0.03) p = 0.306 |
| Between-Person: Cannabis Intercept with | | | | |
| Internalizing Intercept | 0.14 (0.08) p = 0.078 | **0.28 (0.06) p < 0.001** | - | **0.23 (0.06) p < 0.001** |
| Cannabis Slope | **-0.34 (0.11) p = 0.002** | **-0.64 (0.09) p < 0.001** | - | **-0.64 (0.09) p < 0.001** |
| Internalizing Slope | 0.01 (0.14) p = 0.961 | -0.05 (0.09) p = 0.586 | - | 0.07 (0.11) p = 0.528 |
| Between-Person: Cannabis Slope with | | | | |
| Internalizing Intercept | -0.01 (0.11) p = 0.910 | -0.08 (0.11) p = 0.469 | - | -0.08 (0.12) p = 0.523 |
| Internalizing Slope | -0.15 (0.25) p = 0.560 | 0.08 (0.20) p = 0.704 | - | -0.03 (0.25) p = 0.892 |
| Between-Person: Internalizing Intercept with | | | | |
| Internalizing Slope | -0.29 (0.09) p = 0.001 | -**0.38 (0.07) p < 0.001** | - | **-0.32 (0.10) p = 0.001** |

Table S10. Time-varying models (fully adjusted; Model 4) with cigarette ASSIST

|  | Depressive Symptoms | | Anxiety Symptoms | |
| --- | --- | --- | --- | --- |
|  | Frequency | Consequences | Frequency | Consequences |
| Within-Person: Cannabis to Internalizing | | | | |
| T1 🡪 T2 | 0.04 (0.02) p = 0.110 | **0.07 (0.03) p = 0.008** | 0.05 (0.03) p = 0.068 | **0.09 (0.03) p = 0.002** |
| T2 🡪 T3 | 0.07 (0.04) p = 0.111 | **0.10 (0.03) p = 0.002** | 0.08 (0.04) p = 0.067 | **0.10 (0.03) p = 0.001** |
| T3 🡪 T4 | 0.06 (0.04) p = 0.113 | **0.10 (0.03) p = 0.002** | 0.08 (0.04) p = 0.064 | **0.11 (0.03) p < 0.001** |
| T4 🡪 T5 | 0.07 (0.05) p = 0.111 | **0.10 (0.04) p = 0.003** | 0.09 (0.05) p = 0.064 | **0.11 (0.03) p = 0.001** |
| T5 🡪 T6 | 0.07 (0.05) p = 0.111 | **0.11 (0.04) p = 0.003** | 0.08 (0.04) p = 0.066 | **0.11 (0.03) p = 0.001** |
| T6 🡪 T7 | 0.07 (0.05) p = 0.113 | **0.11 (0.04) p = 0.002** | 0.09 (0.05) p = 0.064 | **0.12 (0.03) p = 0.001** |
| Within-Person: Internalizing to Cannabis | | | | |
| T1 🡪 T2 | -0.03 (0.05) p = 0.518 | **0.09 (0.03) p = 0.004** | -0.03 (0.04) p = 0.480 | **0.08 (0.03) p = 0.010** |
| T2 🡪 T3 | -0.03 (0.04) p = 0.516 | **0.08 (0.03) p = 0.005** | -0.03 (0.04) p = 0.479 | **0.07 (0.03) p = 0.010** |
| T3 🡪 T4 | -0.03 (0.04) p = 0.515 | **0.08 (0.03) p = 0.004** | -0.03 (0.04) p = 0.477 | **0.08 (0.03) p = 0.010** |
| T4 🡪 T5 | -0.03 (0.05) p = 0.510 | **0.09 (0.03) p = 0.005** | -0.04 (0.05) p = 0.473 | **0.09 (0.04) p = 0.010** |
| T5 🡪 T6 | -0.03 (0.05) p = 0.516 | **0.08 (0.03) p = 0.003** | -0.03 (0.04) p = 0.478 | **0.08 (0.03) p = 0.009** |
| T6 🡪 T7 | -0.03 (0.05) p = 0.514 | **0.10 (0.03) p = 0.003** | -0.03 (0.05) p = 0.478 | **0.10 (0.04) p = 0.008** |
| Between-Person: Cannabis Intercept with | | | | |
| Internalizing Intercept | 0.16 (0.07) p = 0.016 | **0.30 (0.04) p < 0.001** | 0.19 (0.07) p = 0.006 | **0.23 (0.05) p < 0.001** |
| Cannabis Slope | -0.35 (0.09) p < 0.001 | **-0.71 (0.03) p < 0.001** | -0.36 (0.09) p < 0.001 | **-0.70 (0.03) p < 0.001** |
| Internalizing Slope | -0.01 (0.12) p = 0.909 | -0.10 (0.08) p = 0.206 | -0.05 (0.14) p = 0.713 | 0.05 (0.12) p = 0.645 |
| Between-Person: Cannabis Slope with | | | | |
| Internalizing Intercept | -0.03 (0.07) p = 0.697 | -0.10 (0.06) p = 0.118 | -0.08 (0.07) p = 0.277 | -0.07 (0.07) p = 0.368 |
| Internalizing Slope | 0.02 (0.15) p = 0.917 | -0.02 (0.12) p = 0.889 | 0.05 (0.17) p = 0.772 | -0.22 (0.22) p = 0.304 |
| Between-Person: Internalizing Intercept with | | | | |
| Internalizing Slope | -0.07 (0.07) p = 0.329 | **-0.37 (0.06) p < 0.001** | -0.30 (0.10) p = 0.002 | **-0.31 (0.11) p = 0.006** |

Table S11. Time-varying models (fully adjusted; Model 4) with income

|  | Depressive Symptoms | | Anxiety Symptoms | |
| --- | --- | --- | --- | --- |
|  | Frequency | Consequences | Frequency | Consequences |
| Within-Person: Cannabis to Internalizing | | | | |
| T1 🡪 T2 | 0.02 (0.04) p = 0.689 | **0.08 (0.03) p = 0.001** | 0.06 (0.04) p = 0.116 | **0.10 (0.03) p < 0.001** |
| T2 🡪 T3 | 0.02 (0.04) p = 0.688 | **0.13 (0.03) p < 0.001** | 0.06 (0.04) p = 0.115 | **0.12 (0.03) p < 0.001** |
| T3 🡪 T4 | 0.01 (0.04) p = 0.688 | **0.13 (0.03) p < 0.001** | 0.05 (0.03) p = 0.113 | **0.13 (0.03) p < 0.001** |
| T4 🡪 T5 | 0.02 (0.04) p = 0.689 | **0.14 (0.03) p < 0.001** | 0.06 (0.04) p = 0.117 | **0.13 (0.03) p < 0.001** |
| T5 🡪 T6 | 0.02 (0.04) p = 0.688 | **0.15 (0.04) p < 0.001** | 0.06 (0.04) p = 0.118 | **0.13 (0.03) p < 0.001** |
| T6 🡪 T7 | 0.02 (0.04) p = 0.688 | **0.15 (0.04) p < 0.001** | 0.06 (0.04) p = 0.116 | **0.14 (0.03) p < 0.001** |
| Within-Person: Internalizing to Cannabis | | | | |
| T1 🡪 T2 | 0.00 (0.03) p = 0.874 | **0.10 (0.03) p = 0.002** | 0.02 (0.03) p = 0.485 | **0.07 (0.03) p = 0.009** |
| T2 🡪 T3 | 0.01 (0.04) p = 0.874 | **0.09 (0.03) p = 0.001** | 0.02 (0.03) p = 0.487 | **0.07 (0.03) p = 0.009** |
| T3 🡪 T4 | 0.01 (0.04) p = 0.874 | **0.09 (0.03) p = 0.001** | 0.03 (0.04) p = 0.487 | **0.08 (0.03) p = 0.010** |
| T4 🡪 T5 | 0.01 (0.04) p = 0.874 | **0.10 (0.03) p = 0.002** | 0.03 (0.04) p = 0.487 | **0.09 (0.04) p = 0.010** |
| T5 🡪 T6 | 0.01 (0.04) p = 0.874 | **0.09 (0.03) p = 0.001** | 0.03 (0.04) p = 0.486 | **0.08 (0.03) p = 0.009** |
| T6 🡪 T7 | 0.01 (0.04) p = 0.874 | **0.11 (0.03) p = 0.001** | 0.03 (0.04) p = 0.485 | **0.10 (0.04) p = 0.008** |
| Between-Person: Cannabis Intercept with | | | | |
| Internalizing Intercept | 0.23 (0.06) p < 0.001 | **0.29 (0.04) p < 0.001** | 0.20 (0.06) p = 0.002 | **0.24 (0.05) p < 0.001** |
| Cannabis Slope | -0.44 (0.07) p < 0.001 | **-0.71 (0.03) p < 0.001** | -0.44 (0.06) p < 0.001 | **-0.70 (0.03) p < 0.001** |
| Internalizing Slope | -0.11 (0.10) p = 0.268 | -0.09 (0.08) p = 0.293 | -0.03 (0.12) p = 0.825 | 0.03 (0.11) p = 0.762 |
| Between-Person: Cannabis Slope with | | | | |
| Internalizing Intercept | -0.11 (0.08) p = 0.158 | -0.09 (0.06) p = 0.161 | -0.11 (0.08) p = 0.175 | -0.08 (0.07) p = 0.258 |
| Internalizing Slope | 0.18 (0.15) p = 0.222 | -0.02 (0.14) p = 0.872 | 0.08 (0.17) p = 0.632 | -0.13 (0.17) p = 0.455 |
| Between-Person: Internalizing Intercept with | | | | |
| Internalizing Slope | -0.34 (0.07) p < 0.001 | -0.33 (0.08) p < 0.001 | -0.30 (0.09) p = 0.002 | -0.30 (0.11) p = 0.004 |

Table S12. Within-time adjustment models (fully adjusted; Model 5) with drinks, cigarettes, and income

|  | Depressive Symptoms | | Anxiety Symptoms | |
| --- | --- | --- | --- | --- |
|  | Frequency | Consequences | Frequency | Consequences |
| Within-Person: Cannabis to Internalizing | | | | |
| T1 🡪 T2 | -0.004 (0.034) p = 0.912 | **0.124 (0.042) p = 0.003** | 0.021 (0.046) p = 0.648 | **0.123 (0.042) p = 0.003** |
| T2 🡪 T3 | -0.004 (0.038) p = 0.912 | **0.13 (0.045) p = 0.004** | 0.019 (0.041) p = 0.649 | **0.117 (0.04) p = 0.003** |
| T3 🡪 T4 | -0.004 (0.036) p = 0.912 | **0.099 (0.034) p = 0.004** | 0.019 (0.041) p = 0.65 | **0.097 (0.031) p = 0.002** |
| T4 🡪 T5 | -0.004 (0.036) p = 0.912 | **0.14 (0.047) p = 0.003** | 0.021 (0.046) p = 0.65 | **0.147 (0.047) p = 0.002** |
| T5 🡪 T6 | -0.004 (0.037) p = 0.912 | **0.107 (0.039) p = 0.006** | 0.019 (0.042) p = 0.647 | **0.1 (0.032) p = 0.002** |
| T6 🡪 T7 | -0.005 (0.048) p = 0.912 | **0.133 (0.049) p = 0.007** | 0.021 (0.047) p = 0.649 | **0.121 (0.043) p = 0.005** |
| Within-Person: Internalizing to Cannabis | | | | |
| T1 🡪 T2 | -0.003 (0.041) p = 0.951 | 0.055 (0.029) p = 0.057 | 0.001 (0.034) p = 0.973 | 0.03 (0.026) p = 0.242 |
| T2 🡪 T3 | -0.003 (0.054) p = 0.951 | 0.072 (0.039) p = 0.066 | 0.001 (0.041) p = 0.973 | 0.037 (0.033) p = 0.262 |
| T3 🡪 T4 | -0.003 (0.055) p = 0.951 | 0.06 (0.033) p = 0.069 | 0.002 (0.049) p = 0.973 | 0.034 (0.03) p = 0.259 |
| T4 🡪 T5 | -0.004 (0.063) p = 0.951 | 0.087 (0.046) p = 0.061 | 0.002 (0.05) p = 0.973 | 0.045 (0.039) p = 0.255 |
| T5 🡪 T6 | -0.003 (0.055) p = 0.951 | 0.065 (0.034) p = 0.054 | 0.001 (0.041) p = 0.973 | 0.031 (0.027) p = 0.255 |
| T6 🡪 T7 | -0.003 (0.055) p = 0.951 | 0.087 (0.048) p = 0.067 | 0.002 (0.047) p = 0.973 | 0.047 (0.043) p = 0.277 |
| Between-Person: Cannabis Intercept with | | | | |
| Internalizing Intercept | **0.112 (0.053) p = 0.035** | **0.202 (0.087) p = 0.021** | 0.072 (0.065) p = 0.268 | 0.117 (0.082) p = 0.154 |
| Cannabis Slope | **-0.542 (0.051) p = 0** | **-0.591 (0.12) p = 0** | **-0.405 (0.095) p = 0** | **-0.588 (0.12) p = 0** |
| Internalizing Slope | -0.061 (0.072) p = 0.398 | -0.09 (0.122) p = 0.463 | -0.028 (0.105) p = 0.793 | 0.003 (0.135) p = 0.983 |
| Between-Person: Cannabis Slope with | | | | |
| Internalizing Intercept | -0.053 (0.057) p = 0.353 | 0.06 (0.122) p = 0.626 | -0.094 (0.086) p = 0.271 | 0.049 (0.129) p = 0.702 |
| Internalizing Slope | 0.106 (0.083) p = 0.198 | -0.075 (0.195) p = 0.7 | 0.25 (0.145) p = 0.086 | 0.006 (0.224) p = 0.979 |
| Between-Person: Internalizing Intercept with | | | | |
| Internalizing Slope | **-0.416 (0.078) p = 0** | **-0.403 (0.106) p = 0** | **-0.387 (0.094) p = 0** | **-0.347 (0.127) p = 0.006** |

1. **Sensitivity Analyses in full sample**

Table S12. Standardized estimates (fully adjusted models) in full sample (N = 1331).

|  | Depressive Symptoms | | Anxiety Symptoms | |
| --- | --- | --- | --- | --- |
|  | Frequency | Consequences | Frequency | Consequences |
| Within-Person: Cannabis to Internalizing | | | | |
| T1 🡪 T2 | -0.02 (0.04) p = 0.555 | **0.08 (0.03) p = 0.016** | 0.00 (0.04) p = 0.991 | **0.07 (0.03) p = 0.040** |
| T2 🡪 T3 | -0.02 (0.04) p = 0.556 | **0.07 (0.03) p = 0.018** | 0.00 (0.04) p = 0.991 | **0.06 (0.03) p = 0.041** |
| T3 🡪 T4 | -0.02 (0.03) p = 0.558 | **0.06 (0.03) p = 0.017** | 0.00 (0.03) p = 0.991 | **0.06 (0.03) p = 0.036** |
| T4 🡪 T5 | -0.02 (0.04) p = 0.557 | **0.07 (0.03) p = 0.019** | 0.00 (0.04) p = 0.991 | **0.06 (0.03) p = 0.038** |
| T5 🡪 T6 | -0.02 (0.04) p = 0.556 | **0.07 (0.03) p = 0.017** | 0.00 (0.04) p = 0.991 | **0.05 (0.03) p = 0.035** |
| T6 🡪 T7 | -0.02 (0.04) p = 0.557 | **0.07 (0.03) p = 0.023** | 0.00 (0.04) p = 0.991 | **0.06 (0.03) p = 0.041** |
| Within-Person: Internalizing to Cannabis | | | | |
| T1 🡪 T2 | 0.00 (0.04) p = 0.918 | **0.04 (0.02) p = 0.037** | 0.03 (0.04) p = 0.502 | 0.03 (0.02) p = 0.196 |
| T2 🡪 T3 | 0.00 (0.05) p = 0.918 | **0.05 (0.02) p = 0.039** | 0.03 (0.04) p = 0.502 | 0.03 (0.02) p = 0.203 |
| T3 🡪 T4 | 0.00 (0.05) p = 0.918 | **0.05 (0.02) p = 0.039** | 0.03 (0.05) p = 0.502 | 0.03 (0.02) p = 0.202 |
| T4 🡪 T5 | 0.00 (0.05) p = 0.918 | **0.06 (0.03) p = 0.038** | 0.04 (0.05) p = 0.503 | 0.03 (0.03) p = 0.200 |
| T5 🡪 T6 | 0.00 (0.05) p = 0.918 | **0.05 (0.02) p = 0.031** | 0.03 (0.05) p = 0.501 | 0.03 (0.02) p = 0.194 |
| T6 🡪 T7 | 0.00 (0.05) p = 0.918 | **0.05 (0.03) p = 0.040** | 0.03 (0.05) p = 0.502 | 0.03 (0.03) p = 0.209 |
| Between-Person: Cannabis Intercept with | | | | |
| Internalizing Intercept | 0.25 (0.04) p < 0.001 | **0.26 (0.05) p < 0.001** | 0.22 (0.04) p < 0.001 | **0.22 (0.05) p < 0.001** |
| Cannabis Slope | -0.52 (0.04) p < 0.001 | **-0.68 (0.08) p < 0.001** | -0.52 (0.04) p < 0.001 | **-0.68 (0.08) p < 0.001** |
| Internalizing Slope | -0.17 (0.07) p = 0.012 | -0.10 (0.08) p = 0.189 | -0.09 (0.08) p = 0.243 | 0.04 (0.10) p = 0.691 |
| Between-Person: Cannabis Slope with | | | | |
| Internalizing Intercept | -0.12 (0.05) p = 0.017 | -0.10 (0.09) p = 0.285 | -0.14 (0.05) p = 0.009 | -0.09 (0.10) p = 0.359 |
| Internalizing Slope | 0.16 (0.10) p = 0.084 | 0.10 (0.16) p = 0.545 | 0.12 (0.10) p = 0.222 | -0.02 (0.21) p = 0.908 |
| Between-Person: Internalizing Intercept with | | | | |
| Internalizing Slope | -0.28 (0.06) p < 0.001 | **-0.33 (0.07) p < 0.001** | -0.20 (0.08) p = 0.010 | **-0.23 (0.10) p = 0.019** |

Table S13. Stratified effects (fully adjusted) based on sex assigned at birth in full (n = 1331) sample (Model 3; n = 729 female)

|  | Depressive Symptoms | | | | Anxiety Symptoms | | | |
| --- | --- | --- | --- | --- | --- | --- | --- | --- |
|  | Frequency | | Consequences | | Frequency | | Consequences | |
|  | Female | Male | Female | Male | Female | Male | Female | Male |
| Within-Person: Cannabis to Internalizing | | | | | | | | |
| T1 🡪 T2 | - | - | **0.12 (0.03) p < 0.001** | -0.08 (0.06) p = 0.153 | - | - | **0.11 (0.03) p = 0.001** | -0.10 (0.06) p = 0.128 |
| T2 🡪 T3 | - | - | **0.12 (0.04) p = 0.001** | -0.07 (0.04) p = 0.114 | - | - | **0.10 (0.03) p = 0.002** | -0.08 (0.05) p = 0.100 |
| T3 🡪 T4 | - | - | **0.11 (0.03) p = 0.001** | -0.06 (0.04) p = 0.126 | - | - | **0.10 (0.03) p = 0.001** | -0.07 (0.05) p = 0.112 |
| T4 🡪 T5 | - | - | **0.13 (0.04) p < 0.001** | -0.06 (0.04) p = 0.108 | - | - | **0.12 (0.04) p = 0.001** | -0.07 (0.04) p = 0.099 |
| T5 🡪 T6 | - | - | **0.13 (0.04) p < 0.001** | -0.06 (0.04) p = 0.128 | - | - | **0.10 (0.03) p = 0.001** | -0.07 (0.04) p = 0.117 |
| T6 🡪 T7 | - | - | **0.14 (0.04) p = 0.001** | -0.05 (0.03) p = 0.100 | - | - | **0.12 (0.04) p = 0.001** | -0.06 (0.04) p = 0.099 |
| Within-Person: Internalizing to Cannabis | | | | | | | | |
| T1 🡪 T2 | - | - | **0.06 (0.03) p = 0.024** | 0.02 (0.03) p = 0.437 | - | - | **0.05 (0.03) p = 0.030** | -0.03 (0.04) p = 0.366 |
| T2 🡪 T3 | - | - | **0.06 (0.03) p = 0.031** | 0.03 (0.03) p = 0.435 | - | - | **0.06 (0.03) p = 0.035** | -0.04 (0.04) p = 0.354 |
| T3 🡪 T4 | - | - | **0.06 (0.03) p = 0.036** | 0.03 (0.04) p = 0.432 | - | - | **0.05 (0.03) p = 0.039** | -0.04 (0.05) p = 0.357 |
| T4 🡪 T5 | - | - | **0.07 (0.03) p = 0.034** | 0.03 (0.04) p = 0.428 | - | - | **0.06 (0.03) p = 0.036** | -0.04 (0.05) p = 0.362 |
| T5 🡪 T6 | - | - | **0.06 (0.03) p = 0.027** | 0.03 (0.04) p = 0.419 | - | - | **0.05 (0.02) p = 0.031** | -0.04 (0.05) p = 0.365 |
| T6 🡪 T7 | - | - | **0.07 (0.03) p = 0.035** | 0.03 (0.04) p = 0.444 | - | - | **0.07 (0.03) p = 0.038** | -0.04 (0.04) p = 0.328 |
| Between-Person: Cannabis Intercept with | | | | | | | | |
| Internalizing Intercept | - | - | **0.35 (0.06) p < 0.001** | **0.25 (0.07) p < 0.001** | - | - | **0.25 (0.07) p < 0.001** | **0.32 (0.08) p < 0.001** |
| Cannabis Slope | - | - | **-0.87 (0.04) p < 0.001** | **-0.55 (0.09) p < 0.001** | - | - | **-0.87 (0.04) p < 0.001** | **-0.55 (0.09) p < 0.001** |
| Internalizing Slope | - | - | -0.25 (0.11) p = 0.023 | -0.17 (0.10) p = 0.067 | - | - | -0.03 (0.13) p = 0.849 | -0.25 (0.19) p = 0.184 |
| Between-Person: Cannabis Slope with | | | | | | | | |
| Internalizing Intercept | - | - | **-0.27 (0.09) p = 0.004** | -0.06 (0.10) p = 0.566 | - | **-** | **-0.20 (0.10) p = 0.041** | -0.12 (0.12) p = 0.323 |
| Internalizing Slope | - | - | 0.27 (0.14) p = 0.054 | 0.32 (0.16) p = 0.040 | - | - | 0.10 (0.18) p = 0.591 | 0.39 (0.29) p = 0.174 |
| Between-Person: Internalizing Intercept with | | | | | | | | |
| Internalizing Slope | - | - | **-0.40 (0.09) p < 0.001** | -0.28 (0.13) p = 0.035 | - | - | **-0.38 (0.09) p < 0.001** | 0.11 (0.34) p = 0.754 |

Table S14. Stratified effects (fully adjusted) based on clinical cut-offs in full (n = 1331) sample (Model 3; PHQ-9: n = 414 above threshold).

|  | Cannabis Consequences & Depressive Symptoms | |
| --- | --- | --- |
|  | Sub-Clinical | Clinical |
| Within-Person: Cannabis to Internalizing | | |
| T1 🡪 T2 | 0.00 (0.04) p < 0.9 | **0.16 (0.05) p = 0.001** |
| T2 🡪 T3 | 0.00 (0.03) p < 0.9 | **0.16 (0.06) p = 0.004** |
| T3 🡪 T4 | 0.00 (0.03) p < 0.9 | **0.15 (0.06) p = 0.007** |
| T4 🡪 T5 | 0.00 (0.03) p < 0.9 | **0.16 (0.06) p = 0.005** |
| T5 🡪 T6 | 0.00 (0.03) p < 0.9 | **0.17 (0.05) p = 0.002** |
| T6 🡪 T7 | 0.00 (0.03) p < 0.9 | **0.19 (0.07) p = 0.005** |
| Within-Person: Internalizing to Cannabis | | |
| T1 🡪 T2 | 0.00 (0.01) p < 0.9 | **0.08 (0.03) p = 0.002** |
| T2 🡪 T3 | 0.00 (0.03) p < 0.9 | **0.11 (0.04) p = 0.005** |
| T3 🡪 T4 | 0.00 (0.03) p < 0.9 | **0.12 (0.04) p = 0.004** |
| T4 🡪 T5 | 0.00 (0.03) p < 0.9 | **0.12 (0.04) p = 0.005** |
| T5 🡪 T6 | 0.00 (0.03) p < 0.9 | **0.10 (0.03) p = 0.003** |
| T6 🡪 T7 | 0.00 (0.03) p < 0.9 | **0.12 (0.04) p = 0.009** |
| Between-Person: Cannabis Intercept with | | |
| Internalizing Intercept | **0.18 (0.06) p = 0.005** | 0.11 (0.12) p = 0.339 |
| Cannabis Slope | **-0.72 (0.08) p = 0.000** | **-0.73 (0.10) p = 0.000** |
| Internalizing Slope | -0.02 (0.07) p = 0.783 | -0.05 (0.14) p = 0.739 |
| Between-Person: Cannabis Slope with | | |
| Internalizing Intercept | -0.13 (0.10) p = 0.178 | 0.01 (0.18) p = 0.956 |
| Internalizing Slope | 0.10 (0.12) p = 0.404 | 0.17 (0.20) p = 0.410 |
| Between-Person: Internalizing Intercept with | | |
| Internalizing Slope | 0.21 (0.16) p = 0.195 | 0.09 (0.28) p = 0.743 |

1. **Exploratory Moderation Effects**

Models were invariant across sexual orientation, adverse childhood experiences, and location. There were significant model differences based on family history (i.e., CAST scores), reported below.

Table S15. Stratified effects (fully adjusted) based on family history (Model 3; n=435 with a CAST score of 1+; n=518 with a CAST score of 0).

|  | Depressive Symptoms | | | | Anxiety Symptoms | | | |
| --- | --- | --- | --- | --- | --- | --- | --- | --- |
|  | Frequency | | Consequences | | Frequency | | Consequences | |
|  | - | + | - | + | - | + | - | + |
| Within-Person: Cannabis to Internalizing | | | | | | | | |
| T1 🡪 T2 | -0.05 (0.06) p = 0.404 | -0.02 (0.05) p = 0.724 | - | - | 0.10 (0.07) p = 0.173 | -0.04 (0.06) p = 0.480 | - | - |
| T2 🡪 T3 | -0.05 (0.06) p = 0.403 | -0.02 (0.05) p = 0.725 | - | - | 0.09 (0.06) p = 0.167 | -0.04 (0.05) p = 0.479 | - | - |
| T3 🡪 T4 | -0.04 (0.05) p = 0.408 | -0.02 (0.05) p = 0.725 | - | - | 0.08 (0.06) p = 0.164 | -0.04 (0.05) p = 0.481 | - | - |
| T4 🡪 T5 | -0.05 (0.06) p = 0.402 | -0.02 (0.05) p = 0.725 | - | - | 0.09 (0.07) p = 0.170 | -0.04 (0.06) p = 0.477 | - | - |
| T5 🡪 T6 | -0.05 (0.06) p = 0.411 | -0.02 (0.06) p = 0.724 | - | - | 0.08 (0.06) p = 0.172 | -0.04 (0.06) p = 0.475 | - | - |
| T6 🡪 T7 | -0.05 (0.06) p = 0.413 | -0.02 (0.06) p = 0.725 | - | - | 0.10 (0.07) p = 0.169 | -0.04 (0.06) p = 0.478 | - | - |
| Within-Person: Internalizing to Cannabis | | | | | | | | |
| T1 🡪 T2 | -0.16 (0.07) p = 0.018 | 0.09 (0.05) p = 0.060 | - | - | -0.05 (0.07) p = 0.438 | 0.06 (0.05) p = 0.222 | - | - |
| T2 🡪 T3 | -0.16 (0.07) p = 0.019 | 0.12 (0.06) p = 0.059 | - | - | -0.05 (0.06) p = 0.442 | 0.09 (0.07) p = 0.232 | - | - |
| T3 🡪 T4 | -0.15 (0.06) p = 0.022 | 0.13 (0.07) p = 0.059 | - | - | -0.05 (0.07) p = 0.434 | 0.10 (0.09) p = 0.226 | - | - |
| T4 🡪 T5 | -0.19 (0.08) p = 0.017 | 0.14 (0.08) p = 0.060 | - | - | -0.06 (0.08) p = 0.435 | 0.11 (0.09) p = 0.229 | - | - |
| T5 🡪 T6 | -0.15 (0.07) p = 0.023 | 0.13 (0.06) p = 0.054 | - | - | -0.05 (0.07) p = 0.439 | 0.09 (0.08) p = 0.221 | - | - |
| T6 🡪 T7 | -0.16 (0.07) p = 0.016 | 0.12 (0.06) p = 0.056 | - | - | -0.06 (0.07) p = 0.437 | 0.09 (0.07) p = 0.222 | - | - |
| Between-Person: Cannabis Intercept with | | | | | | | | |
| Internalizing Intercept | 0.30 (0.06) p < 0.001 | 0.14 (0.07) p = 0.039 | - | - | 0.27 (0.07) p < 0.001 | 0.11 (0.07) p = 0.131 | - | - |
| Cannabis Slope | -0.53 (0.06) p < 0.001 | -0.60 (0.05) p < 0.001 | - | - | -0.53 (0.06) p < 0.001 | -0.60 (0.05) p < 0.001 | - | - |
| Internalizing Slope | -0.22 (0.11) p = 0.054 | -0.02 (0.10) p = 0.849 | - | - | -0.17 (0.11) p = 0.114 | 0.13 (0.12) p = 0.267 | - | - |
| Between-Person: Cannabis Slope with | | | | | | | | |
| Internalizing Intercept | -0.10 (0.07) p = 0.181 | -0.12 (0.08) p = 0.121 | - | - | -0.09 (0.07) p = 0.213 | -0.10 (0.08) p = 0.236 | - | - |
| Internalizing Slope | 0.16 (0.13) p = 0.210 | 0.17 (0.13) p = 0.176 | - | - | 0.10 (0.12) p = 0.396 | 0.04 (0.14) p = 0.783 | - | - |
| Between-Person: Internalizing Intercept with | | | | | | | | |
| Internalizing Slope | -0.23 (0.10) p = 0.017 | -0.52 (0.08) p < 0.001 | - | - | -0.19 (0.09) p = 0.026 | -0.52 (0.10) p < 0.001 | - | - |

1. **Cannabis Consequences Sensitivity Analysis**

For the sensitivity analysis, we dropped the following items from the Brief MACQ:

We dropped:

1. I have been unhappy because of my marijuana use.
2. I have had trouble sleeping after stopping or cutting down on marijuana use.
3. I haven’t been as sharp mentally because of my marijuana use.
4. I have felt anxious, irritable, lost my appetite or had stomach pains after stopping or cutting down on marijuana
5. I have had less energy or felt tired because of my marijuana use.
6. I have lost motivation to do things because of my marijuana use.

We retained:

1. The quality of my work or schoolwork has suffered because of my marijuana use.
2. I have driven a car when I was high.
3. I have felt in a fog, sluggish, tired, or dazed the morning after using marijuana.
4. I have gotten into physical fights because of my marijuana use.
5. I have spent too much time using marijuana.
6. I have felt like I needed a hit of marijuana after I’d gotten up (that is, before breakfast).
7. I have become very rude, obnoxious, or insulting after using marijuana.
8. I have been less physically active because of my marijuana use.
9. I have neglected obligations to family, work, or school because of my marijuana use.
10. When using marijuana I have done impulsive things that I regretted later.
11. I have awakened the day after using marijuana and found I could not remember a part of the evening before
12. I have been overweight because of my marijuana use.
13. I have received a lower grade on an exam or paper than I ordinarily could have because of marijuana use.
14. I have tried to quit using marijuana because I thought I was using too much.
15. I often have thought about needing to cut down or to stop using marijuana.

Table 16. Fully-adjusted models with brief-MACQ items dropped that map onto internalizing symptomatology (n = 15 items in total).

|  | Cannabis-Related Consequences | | |
| --- | --- | --- | --- |
|  | Anxiety | Depression | |
| Within-Person: Cannabis to Internalizing | | |  |
| T1 🡪 T2 | **0.06 (0.03) p = 0.048** | 0.06 (0.03) p = 0.071 | |
| T2 🡪 T3 | **0.06 (0.03) p = 0.049** | 0.06 (0.03) p = 0.073 | |
| T3 🡪 T4 | **0.06 (0.03) p = 0.044** | 0.05 (0.03) p = 0.072 | |
| T4 🡪 T5 | **0.06 (0.03) p = 0.046** | 0.06 (0.03) p = 0.078 | |
| T5 🡪 T6 | **0.06 (0.03) p = 0.043** | 0.05 (0.03) p = 0.074 | |
| T6 🡪 T7 | **0.06 (0.03) p = 0.049** | 0.06 (0.03) p = 0.083 | |
| Within-Person: Internalizing to Cannabis | | |  |
| T1 🡪 T2 | 0.03 (0.02) p = 0.248 | **0.06 (0.03) p = 0.033** | |
| T2 🡪 T3 | 0.03 (0.03) p = 0.253 | **0.06 (0.03) p = 0.034** | |
| T3 🡪 T4 | 0.03 (0.03) p = 0.253 | **0.06 (0.03) p = 0.034** | |
| T4 🡪 T5 | 0.04 (0.03) p = 0.250 | **0.07 (0.03) p = 0.032** | |
| T5 🡪 T6 | 0.03 (0.03) p = 0.245 | **0.06 (0.03) p = 0.026** | |
| T6 🡪 T7 | 0.04 (0.03) p = 0.260 | **0.06 (0.03) p = 0.036** | |
| Between-Person: Cannabis Intercept with | | |  |
| Internalizing Intercept | **0.21 (0.06) p < 0.001** | **0.26 (0.05) p < 0.001** | |
| Cannabis Slope | **-0.69 (0.06) p < 0.001** | **-0.69 (0.06) p < 0.001** | |
| Internalizing Slope | 0.06 (0.11) p = 0.558 | -0.07 (0.09) p = 0.434 | |
| Between-Person: Cannabis Slope with | | |  |
| Internalizing Intercept | -0.06 (0.10) p = 0.516 | -0.10 (0.09) p = 0.240 | |
| Internalizing Slope | -0.03 (0.20) p = 0.874 | 0.12 (0.16) p = 0.465 | |
| Between-Person: Internalizing Intercept with | | |  |
| Internalizing Slope | **-0.33 (0.10) p = 0.001** | **-0.38 (0.07) p < 0.001** | |

Additional Notes about Models:

- Some models required constraints of residual variance which were Heywood cases (small negative variance close to zero).

- Cannabis frequency LCM-SR stratified successfully ran once constraining small negative variances. However, cannabis frequency with depression stratified by sexual orientation did not converge despite trying several different methods to resolve.

- Time-varying models:

|  | Cann Freq  & GAD | Cann Freq  & PHQ | MACQ & GAD | MACQ & PHQ |
| --- | --- | --- | --- | --- |
| Drinks | No Convergence (even with removal of alcohol slope) | Convergence (with removal of alcohol slope) | Converged | Converged |
| Cigs | Converged | Converged | Converged | Converged |
| Income | Converged | Converged | Converged | Converged |
