# Supplementary material for: Examining bidirectional associations between cannabis use and internalizing symptoms among high-risk emerging adults: A prospective cohort study
Source: Psychol Med. 2025 Oct 3;55:e291. doi: 10.1017/S0033291725101700 (PMC12527518; doi:10.1017/S0033291725101700)
Supplement: Halladay et al. supplementary material 2 — Halladay et al. supplementary material [file S0033291725101700sup002.docx]

**Examining bidirectional associations between cannabis use and internalizing symptoms among high-risk emerging adults: A prospective cohort study**

**SUPPLEMENTARY MATERIALS – EXAMPLE CODE**

**TITLE: LCM-SR MACQ & PHQ-9 Power Analysis (Part 1)**

DATA: FILE IS Data_N961-V.csv;

VARIABLE:

NAMES = ID sample age sex race orient

income degree phq gad ace trauma

cast famhist drks drug combvape cig ecig ftnd dudit

cigv1 cigv2 cigv3 cigv4 cigv5 cigv6 cigv7

drinks1 drinks2 drinks3 drinks4 drinks5 drinks6 drinks7

inc1 inc2 inc3 inc4 inc5 inc6 inc7

cann1 cann2 cann3 cann4 cann5 cann6 cann7

macq1 macq2 macq3 macq4 macq5 macq6 macq7

phq1 phq2 phq3 phq4 phq5 phq6 phq7

gad1 gad2 gad3 gad4 gad5 gad6 gad7;

USEVARIABLES = macq1 macq2 macq3 macq4 macq5 macq6 macq7

phq1 phq2 phq3 phq4 phq5 phq6 phq7;

IDVARIABLE = ID;

MISSING = .;

ANALYSIS:

MODEL = NOCOV;

ESTIMATOR = MLR;

MODEL:

!! Between components (I, S)

RI_X BY macq1@1 macq2@1 macq3@1 macq4@1 macq5@1 macq6@1 macq7@1;

RS_X BY macq1@0 macq2@1 macq3@2 macq4@3 macq5@4 macq6@5 macq7@6;

RI_Y BY phq1@1 phq2@1 phq3@1 phq4@1 phq5@1 phq6@1 phq7@1;

RS_Y BY phq1@0 phq2@1 phq3@2 phq4@3 phq5@4 phq6@5 phq7@6;

!! Within-person centered variables

Wx1 BY macq1@1;

Wx2 BY macq2@1;

Wx3 BY macq3@1;

Wx4 BY macq4@1;

Wx5 BY macq5@1;

Wx6 BY macq6@1;

Wx7 BY macq7@1;

Wy1 BY phq1@1;

Wy2 BY phq2@1;

Wy3 BY phq3@1;

Wy4 BY phq4@1;

Wy5 BY phq5@1;

Wy6 BY phq6@1;

Wy7 BY phq7@1;

macq1-macq7@0; !! Constrain Measurement Error

phq1-phq7@0; !! Constrain Measurement Error

!! Auto- and cross-lagged effects

Wx2 ON Wx1 (xx);

Wx2 ON Wy1 (yx);

Wy2 ON Wx1 (xy);

Wy2 ON Wy1 (yy);

Wx3 ON Wx2 (xx);

Wx3 ON Wy2 (yx);

Wy3 ON Wx2 (xy);

Wy3 ON Wy2 (yy);

Wx4 ON Wx3 (xx);

Wx4 ON Wy3 (yx);

Wy4 ON Wx3 (xy);

Wy4 ON Wy3 (yy);

Wx5 ON Wx4 (xx);

Wx5 ON Wy4 (yx);

Wy5 ON Wx4 (xy);

Wy5 ON Wy4 (yy);

Wx6 ON Wx5 (xx);

Wx6 ON Wy5 (yx);

Wy6 ON Wx5 (xy);

Wy6 ON Wy5 (yy);

Wx7 ON Wx6 (xx);

Wx7 ON Wy6 (yx);

Wy7 ON Wx6 (xy);

Wy7 ON Wy6 (yy);

!! Covariances between growth factors

RI_X WITH RI_Y RS_X RS_Y;

RS_X WITH RI_Y RS_Y;

RI_Y WITH RS_Y;

!! Covariances

Wx1 WITH Wy1;

Wx2 WITH Wy2;

Wx3 WITH Wy3;

Wx4 WITH Wy4;

Wx5 WITH Wy5;

Wx6 WITH Wy6;

Wx7 WITH Wy7;

OUTPUT: STDYX;

SAVEDATA: ESTIMATES = LCMSR_MACQ_PHQ_Power.dat;

**TITLE: LCM-SR MACQ & PHQ-9 Power Analysis (Part 2)**

MONTECARLO:

SAVE = LCMSR_MACQ_PHQ_Power.csv;

NAMES = macq1 macq2 macq3 macq4 macq5 macq6 macq7

phq1 phq2 phq3 phq4 phq5 phq6 phq7;

NOBSERVATIONS = 961;

NREPS = 50;

POPULATION = LCMSR_MACQ_PHQ_Power.dat;

COVERAGE = LCMSR_MACQ_PHQ_Power.dat;

ANALYSIS:

MODEL = NOCOV;

ESTIMATOR = MLR;

MODEL POPULATION:

!! Between components (I, S)

RI_X BY macq1@1 macq2@1 macq3@1 macq4@1 macq5@1 macq6@1 macq7@1;

RS_X BY macq1@0 macq2@1 macq3@2 macq4@3 macq5@4 macq6@5 macq7@6;

RI_Y BY phq1@1 phq2@1 phq3@1 phq4@1 phq5@1 phq6@1 phq7@1;

RS_Y BY phq1@0 phq2@1 phq3@2 phq4@3 phq5@4 phq6@5 phq7@6;

!! Within-person centered variables

Wx1 BY macq1@1;

Wx2 BY macq2@1;

Wx3 BY macq3@1;

Wx4 BY macq4@1;

Wx5 BY macq5@1;

Wx6 BY macq6@1;

Wx7 BY macq7@1;

Wy1 BY phq1@1;

Wy2 BY phq2@1;

Wy3 BY phq3@1;

Wy4 BY phq4@1;

Wy5 BY phq5@1;

Wy6 BY phq6@1;

Wy7 BY phq7@1;

macq1-macq7@0; !! Constrain Measurement Error

phq1-phq7@0; !! Constrain Measurement Error

!! Auto- and cross-lagged effects

Wx2 ON Wx1 (xx);

Wx2 ON Wy1 (yx);

Wy2 ON Wx1 (xy);

Wy2 ON Wy1 (yy);

Wx3 ON Wx2 (xx);

Wx3 ON Wy2 (yx);

Wy3 ON Wx2 (xy);

Wy3 ON Wy2 (yy);

Wx4 ON Wx3 (xx);

Wx4 ON Wy3 (yx);

Wy4 ON Wx3 (xy);

Wy4 ON Wy3 (yy);

Wx5 ON Wx4 (xx);

Wx5 ON Wy4 (yx);

Wy5 ON Wx4 (xy);

Wy5 ON Wy4 (yy);

Wx6 ON Wx5 (xx);

Wx6 ON Wy5 (yx);

Wy6 ON Wx5 (xy);

Wy6 ON Wy5 (yy);

Wx7 ON Wx6 (xx);

Wx7 ON Wy6 (yx);

Wy7 ON Wx6 (xy);

Wy7 ON Wy6 (yy);

!! Covariances between growth factors

RI_X WITH RI_Y RS_X RS_Y;

RS_X WITH RI_Y RS_Y;

RI_Y WITH RS_Y;

!! Covariances

Wx1 WITH Wy1;

Wx2 WITH Wy2;

Wx3 WITH Wy3;

Wx4 WITH Wy4;

Wx5 WITH Wy5;

Wx6 WITH Wy6;

Wx7 WITH Wy7;

MODEL:

!! Between components (I, S)

RI_X BY macq1@1 macq2@1 macq3@1 macq4@1 macq5@1 macq6@1 macq7@1;

RS_X BY macq1@0 macq2@1 macq3@2 macq4@3 macq5@4 macq6@5 macq7@6;

RI_Y BY phq1@1 phq2@1 phq3@1 phq4@1 phq5@1 phq6@1 phq7@1;

RS_Y BY phq1@0 phq2@1 phq3@2 phq4@3 phq5@4 phq6@5 phq7@6;

!! Within-person centered variables

Wx1 BY macq1@1;

Wx2 BY macq2@1;

Wx3 BY macq3@1;

Wx4 BY macq4@1;

Wx5 BY macq5@1;

Wx6 BY macq6@1;

Wx7 BY macq7@1;

Wy1 BY phq1@1;

Wy2 BY phq2@1;

Wy3 BY phq3@1;

Wy4 BY phq4@1;

Wy5 BY phq5@1;

Wy6 BY phq6@1;

Wy7 BY phq7@1;

macq1-macq7@0; !! Constrain Measurement Error

phq1-phq7@0; !! Constrain Measurement Error

!! Auto- and cross-lagged effects

Wx2 ON Wx1 (xx);

Wx2 ON Wy1 (yx);

Wy2 ON Wx1 (xy);

Wy2 ON Wy1 (yy);

Wx3 ON Wx2 (xx);

Wx3 ON Wy2 (yx);

Wy3 ON Wx2 (xy);

Wy3 ON Wy2 (yy);

Wx4 ON Wx3 (xx);

Wx4 ON Wy3 (yx);

Wy4 ON Wx3 (xy);

Wy4 ON Wy3 (yy);

Wx5 ON Wx4 (xx);

Wx5 ON Wy4 (yx);

Wy5 ON Wx4 (xy);

Wy5 ON Wy4 (yy);

Wx6 ON Wx5 (xx);

Wx6 ON Wy5 (yx);

Wy6 ON Wx5 (xy);

Wy6 ON Wy5 (yy);

Wx7 ON Wx6 (xx);

Wx7 ON Wy6 (yx);

Wy7 ON Wx6 (xy);

Wy7 ON Wy6 (yy);

!! Covariances between growth factors

RI_X WITH RI_Y RS_X RS_Y;

RS_X WITH RI_Y RS_Y;

RI_Y WITH RS_Y;

!! Covariances

Wx1 WITH Wy1;

Wx2 WITH Wy2;

Wx3 WITH Wy3;

Wx4 WITH Wy4;

Wx5 WITH Wy5;

Wx6 WITH Wy6;

Wx7 WITH Wy7;

OUTPUT: TECH9;

**TITLE: LCMSR with Cannabis-Consequences (MACQ) and Depression (PHQ-9) - All Covariates**

DATA: FILE IS Data_N961-V.csv;

VARIABLE:

NAMES = ID sample age sex race orient

income degree phq gad ace trauma

cast famhist drks drug combvape cig ecig ftnd dudit

cigv1 cigv2 cigv3 cigv4 cigv5 cigv6 cigv7

drinks1 drinks2 drinks3 drinks4 drinks5 drinks6 drinks7

inc1 inc2 inc3 inc4 inc5 inc6 inc7

cann1 cann2 cann3 cann4 cann5 cann6 cann7

macq1 macq2 macq3 macq4 macq5 macq6 macq7

phq1 phq2 phq3 phq4 phq5 phq6 phq7

gad1 gad2 gad3 gad4 gad5 gad6 gad7;

USEVARIABLES = macq1 macq2 macq3 macq4 macq5 macq6 macq7

phq1 phq2 phq3 phq4 phq5 phq6 phq7

age sex race orient degree ace cast;

IDVARIABLE = ID;

MISSING = .;

ANALYSIS:

MODEL = NOCOV;

ESTIMATOR = MLR;

MODEL:

!! Between components (I, S)

RI_X BY macq1@1 macq2@1 macq3@1 macq4@1 macq5@1 macq6@1 macq7@1;

RS_X BY macq1@0 macq2@1 macq3@2 macq4@3 macq5@4 macq6@5 macq7@6;

RI_Y BY phq1@1 phq2@1 phq3@1 phq4@1 phq5@1 phq6@1 phq7@1;

RS_Y BY phq1@0 phq2@1 phq3@2 phq4@3 phq5@4 phq6@5 phq7@6;

!! Within-person centered variables

Wx1 BY macq1@1;

Wx2 BY macq2@1;

Wx3 BY macq3@1;

Wx4 BY macq4@1;

Wx5 BY macq5@1;

Wx6 BY macq6@1;

Wx7 BY macq7@1;

Wy1 BY phq1@1;

Wy2 BY phq2@1;

Wy3 BY phq3@1;

Wy4 BY phq4@1;

Wy5 BY phq5@1;

Wy6 BY phq6@1;

Wy7 BY phq7@1;

macq1-macq7@0; !! Constrain Measurement Error

phq1-phq7@0; !! Constrain Measurement Error

!! Auto- and cross-lagged effects

Wx2 ON Wx1 (xx);

Wx2 ON Wy1 (yx);

Wy2 ON Wx1 (xy);

Wy2 ON Wy1 (yy);

Wx3 ON Wx2 (xx);

Wx3 ON Wy2 (yx);

Wy3 ON Wx2 (xy);

Wy3 ON Wy2 (yy);

Wx4 ON Wx3 (xx);

Wx4 ON Wy3 (yx);

Wy4 ON Wx3 (xy);

Wy4 ON Wy3 (yy);

Wx5 ON Wx4 (xx);

Wx5 ON Wy4 (yx);

Wy5 ON Wx4 (xy);

Wy5 ON Wy4 (yy);

Wx6 ON Wx5 (xx);

Wx6 ON Wy5 (yx);

Wy6 ON Wx5 (xy);

Wy6 ON Wy5 (yy);

Wx7 ON Wx6 (xx);

Wx7 ON Wy6 (yx);

Wy7 ON Wx6 (xy);

Wy7 ON Wy6 (yy);

!! Covariates

RI_X RS_X ON age sex race orient degree ace cast;

RI_Y RS_Y ON age sex race orient degree ace cast;

!! Covariances between growth factors

RI_X WITH RI_Y RS_X RS_Y;

RS_X WITH RI_Y RS_Y;

RI_Y WITH RS_Y;

!! Covariances

Wx1 WITH Wy1;

Wx2 WITH Wy2;

Wx3 WITH Wy3;

Wx4 WITH Wy4;

Wx5 WITH Wy5;

Wx6 WITH Wy6;

Wx7 WITH Wy7;

OUTPUT: STDYX;

**TITLE: LCM-SR MACQ & PHQ Stratified by Sex**

DATA: FILE IS Data_N961-V.csv;

VARIABLE:

NAMES = ID sample age sex race orient

income degree phq gad ace trauma

cast famhist drks drug combvape cig ecig ftnd dudit

cigv1 cigv2 cigv3 cigv4 cigv5 cigv6 cigv7

drinks1 drinks2 drinks3 drinks4 drinks5 drinks6 drinks7

inc1 inc2 inc3 inc4 inc5 inc6 inc7

cann1 cann2 cann3 cann4 cann5 cann6 cann7

macq1 macq2 macq3 macq4 macq5 macq6 macq7

phq1 phq2 phq3 phq4 phq5 phq6 phq7

gad1 gad2 gad3 gad4 gad5 gad6 gad7;

USEVARIABLES = macq1 macq2 macq3 macq4 macq5 macq6 macq7

phq1 phq2 phq3 phq4 phq5 phq6 phq7;

IDVARIABLE = ID;

GROUPING = sex (0 = REFERENCE 1 = NONREFERENCE);

MISSING = .;

ANALYSIS:

MODEL = NOCOV;

ESTIMATOR = MLR;

MODEL:

!! Between components (I, S)

RI_X BY macq1@1 macq2@1 macq3@1 macq4@1 macq5@1 macq6@1 macq7@1;

RS_X BY macq1@0 macq2@1 macq3@2 macq4@3 macq5@4 macq6@5 macq7@6;

RI_Y BY phq1@1 phq2@1 phq3@1 phq4@1 phq5@1 phq6@1 phq7@1;

RS_Y BY phq1@0 phq2@1 phq3@2 phq4@3 phq5@4 phq6@5 phq7@6;

!! Within-person centered variables

Wx1 BY macq1@1;

Wx2 BY macq2@1;

Wx3 BY macq3@1;

Wx4 BY macq4@1;

Wx5 BY macq5@1;

Wx6 BY macq6@1;

Wx7 BY macq7@1;

Wy1 BY phq1@1;

Wy2 BY phq2@1;

Wy3 BY phq3@1;

Wy4 BY phq4@1;

Wy5 BY phq5@1;

Wy6 BY phq6@1;

Wy7 BY phq7@1;

macq1-macq7@0; !! Constrain Measurement Error

phq1-phq7@0; !! Constrain Measurement Error

!! Auto- and cross-lagged effects

Wx2 ON Wx1 (xx0);

Wx2 ON Wy1 (yx0);

Wy2 ON Wx1 (xy0);

Wy2 ON Wy1 (yy0);

Wx3 ON Wx2 (xx0);

Wx3 ON Wy2 (yx0);

Wy3 ON Wx2 (xy0);

Wy3 ON Wy2 (yy0);

Wx4 ON Wx3 (xx0);

Wx4 ON Wy3 (yx0);

Wy4 ON Wx3 (xy0);

Wy4 ON Wy3 (yy0);

Wx5 ON Wx4 (xx0);

Wx5 ON Wy4 (yx0);

Wy5 ON Wx4 (xy0);

Wy5 ON Wy4 (yy0);

Wx6 ON Wx5 (xx0);

Wx6 ON Wy5 (yx0);

Wy6 ON Wx5 (xy0);

Wy6 ON Wy5 (yy0);

Wx7 ON Wx6 (xx0);

Wx7 ON Wy6 (yx0);

Wy7 ON Wx6 (xy0);

Wy7 ON Wy6 (yy0);

!! Covariances between growth factors

RI_X WITH RI_Y RS_X RS_Y;

RS_X WITH RI_Y RS_Y;

RI_Y WITH RS_Y;

!! Covariances

Wx1 WITH Wy1;

Wx2 WITH Wy2;

Wx3 WITH Wy3;

Wx4 WITH Wy4;

Wx5 WITH Wy5;

Wx6 WITH Wy6;

Wx7 WITH Wy7;

MODEL NONREFERENCE:

!! Overrule equal constraints across groups

[macq1-macq7];

[phq1-phq7];

[Wx1-Wy7@0];

[RI_X@0 RS_X@0 RI_Y@0 RS_Y@0];

!! Auto- and cross-lagged effects

Wx2 ON Wx1 (xx1);

Wx2 ON Wy1 (yx1);

Wy2 ON Wx1 (xy1);

Wy2 ON Wy1 (yy1);

Wx3 ON Wx2 (xx1);

Wx3 ON Wy2 (yx1);

Wy3 ON Wx2 (xy1);

Wy3 ON Wy2 (yy1);

Wx4 ON Wx3 (xx1);

Wx4 ON Wy3 (yx1);

Wy4 ON Wx3 (xy1);

Wy4 ON Wy3 (yy1);

Wx5 ON Wx4 (xx1);

Wx5 ON Wy4 (yx1);

Wy5 ON Wx4 (xy1);

Wy5 ON Wy4 (yy1);

Wx6 ON Wx5 (xx1);

Wx6 ON Wy5 (yx1);

Wy6 ON Wx5 (xy1);

Wy6 ON Wy5 (yy1);

Wx7 ON Wx6 (xx1);

Wx7 ON Wy6 (yx1);

Wy7 ON Wx6 (xy1);

Wy7 ON Wy6 (yy1);

OUTPUT: STDYX TECH4;

**TITLE: LCM-SR MACQ & PHQ Stratified by Sex (Constrained Equal)**

DATA: FILE IS Data_N961-V.csv;

VARIABLE:

NAMES = ID sample age sex race orient

income degree phq gad ace trauma

cast famhist drks drug combvape cig ecig ftnd dudit

cigv1 cigv2 cigv3 cigv4 cigv5 cigv6 cigv7

drinks1 drinks2 drinks3 drinks4 drinks5 drinks6 drinks7

inc1 inc2 inc3 inc4 inc5 inc6 inc7

cann1 cann2 cann3 cann4 cann5 cann6 cann7

macq1 macq2 macq3 macq4 macq5 macq6 macq7

phq1 phq2 phq3 phq4 phq5 phq6 phq7

gad1 gad2 gad3 gad4 gad5 gad6 gad7;

USEVARIABLES = macq1 macq2 macq3 macq4 macq5 macq6 macq7

phq1 phq2 phq3 phq4 phq5 phq6 phq7;

IDVARIABLE = ID;

GROUPING = sex (0 = REFERENCE 1 = NONREFERENCE);

MISSING = .;

ANALYSIS:

MODEL = NOCOV;

ESTIMATOR = MLR;

MODEL:

!! Between components (I, S)

RI_X BY macq1@1 macq2@1 macq3@1 macq4@1 macq5@1 macq6@1 macq7@1;

RS_X BY macq1@0 macq2@1 macq3@2 macq4@3 macq5@4 macq6@5 macq7@6;

RI_Y BY phq1@1 phq2@1 phq3@1 phq4@1 phq5@1 phq6@1 phq7@1;

RS_Y BY phq1@0 phq2@1 phq3@2 phq4@3 phq5@4 phq6@5 phq7@6;

!! Within-person centered variables

Wx1 BY macq1@1;

Wx2 BY macq2@1;

Wx3 BY macq3@1;

Wx4 BY macq4@1;

Wx5 BY macq5@1;

Wx6 BY macq6@1;

Wx7 BY macq7@1;

Wy1 BY phq1@1;

Wy2 BY phq2@1;

Wy3 BY phq3@1;

Wy4 BY phq4@1;

Wy5 BY phq5@1;

Wy6 BY phq6@1;

Wy7 BY phq7@1;

macq1-macq7@0; !! Constrain Measurement Error

phq1-phq7@0; !! Constrain Measurement Error

!! Auto- and cross-lagged effects

Wx2 ON Wx1 (xx);

Wx2 ON Wy1 (yx);

Wy2 ON Wx1 (xy);

Wy2 ON Wy1 (yy);

Wx3 ON Wx2 (xx);

Wx3 ON Wy2 (yx);

Wy3 ON Wx2 (xy);

Wy3 ON Wy2 (yy);

Wx4 ON Wx3 (xx);

Wx4 ON Wy3 (yx);

Wy4 ON Wx3 (xy);

Wy4 ON Wy3 (yy);

Wx5 ON Wx4 (xx);

Wx5 ON Wy4 (yx);

Wy5 ON Wx4 (xy);

Wy5 ON Wy4 (yy);

Wx6 ON Wx5 (xx);

Wx6 ON Wy5 (yx);

Wy6 ON Wx5 (xy);

Wy6 ON Wy5 (yy);

Wx7 ON Wx6 (xx);

Wx7 ON Wy6 (yx);

Wy7 ON Wx6 (xy);

Wy7 ON Wy6 (yy);

!! Covariances between growth factors

RI_X WITH RI_Y RS_X RS_Y;

RS_X WITH RI_Y RS_Y;

RI_Y WITH RS_Y;

!! Covariances

Wx1 WITH Wy1;

Wx2 WITH Wy2;

Wx3 WITH Wy3;

Wx4 WITH Wy4;

Wx5 WITH Wy5;

Wx6 WITH Wy6;

Wx7 WITH Wy7;

MODEL NONREFERENCE:

!! Overrule equal constraints across groups

[macq1-macq7];

[phq1-phq7];

[Wx1-Wy7@0];

[RI_X@0 RS_X@0 RI_Y@0 RS_Y@0];

OUTPUT: STDYX TECH4;
